# Electrophysiological Profile Remodeling *via* Selective Suppression of Voltage-Gated Currents by *CLN1*/PPT1 Overexpression in Human Neuronal-Like Cells

**DOI:** 10.3389/fncel.2020.569598

**Published:** 2020-12-16

**Authors:** Gian Carlo Demontis, Francesco Pezzini, Elisa Margari, Marzia Bianchi, Biancamaria Longoni, Stefano Doccini, Maciej Maurycy Lalowski, Filippo Maria Santorelli, Alessandro Simonati

**Affiliations:** ^1^Department of Pharmacy, University of Pisa, Pisa, Italy; ^2^Neurology (Child Neurology and Neuropathology), Department of Neuroscience, Biomedicine and Movement, University of Verona, Verona, Italy; ^3^Research Unit for Multi-factorial Diseases, Obesity and Diabetes, Bambino Gesù Children's Hospital Istituto di Ricerca e Cura a Carattere Scientifico, Rome, Italy; ^4^Department of Translational Research and New Technology in Medicine, University of Pisa, Pisa, Italy; ^5^Molecular Medicine for Neurodegenerative and Neuromuscular Diseases Unit, Istituto di Ricerca e Cura a Carattere Scientifico Stella Maris Foundation, Pisa, Italy; ^6^Medicum, Biochemistry/Developmental Biology and HiLIFE-Helsinki Institute of Life Science, Meilahti Clinical Proteomics Core Facility, University of Helsinki, Helsinki, Finland

**Keywords:** differentiated SH-SY5Y neuroblastoma cells, *CLN1*/PPT1 overexpression, transcriptomics, bioinformatics, patch-clamp recording, calcium imaging, voltage-gated ion channels, CLN1 disease

## Abstract

CLN1 disease (OMIM #256730) is an inherited neurological disorder of early childhood with epileptic seizures and premature death. It is associated with mutations in *CLN1* coding for Palmitoyl-Protein Thioesterase 1 (PPT1), a lysosomal enzyme which affects the recycling and degradation of lipid-modified (S-acylated) proteins by removing palmitate residues. Transcriptomic evidence from a neuronal-like cellular model derived from differentiated SH-SY5Y cells disclosed the potential negative roles of *CLN1* overexpression, affecting the elongation of neuronal processes and the expression of selected proteins of the synaptic region. Bioinformatic inquiries of transcriptomic data pinpointed a dysregulated expression of several genes coding for proteins related to voltage-gated ion channels, including subunits of calcium and potassium channels (VGCC and VGKC). In SH-SY5Y cells overexpressing *CLN1* (SH-*CLN1* cells), the resting potential and the membrane conductance in the range of voltages close to the resting potential were not affected. However, patch-clamp recordings indicated a reduction of Ba^2+^ currents through VGCC of SH-*CLN1* cells; Ca^2+^ imaging revealed reduced Ca^2+^ influx in the same cellular setting. The results of the biochemical and morphological investigations of CACNA2D2/α_2_δ-2, an accessory subunit of VGCC, were in accordance with the downregulation of the corresponding gene and consistent with the hypothesis that a lower number of functional channels may reach the plasma membrane. The combined use of 4-AP and NS-1643, two drugs with opposing effects on K_v_11 and K_v_12 subfamilies of VGKC coded by the *KCNH* gene family, provides evidence for reduced functional K_v_12 channels in SH-*CLN1* cells, consistent with transcriptomic data indicating the downregulation of *KCNH4*. The lack of compelling evidence supporting the palmitoylation of many ion channels subunits investigated in this study stimulates inquiries about the role of PPT1 in the trafficking of channels to the plasma membrane. Altogether, these results indicate a reduction of functional voltage-gated ion channels in response to *CLN1*/PPT1 overexpression in differentiated SH-SY5Y cells and provide new insights into the altered neuronal excitability which may underlie the severe epileptic phenotype of CLN1 disease. It remains to be shown if remodeling of such functional channels on plasma membrane can occur as a downstream effect of CLN1 disease.

## Introduction

Mutations in *CLN1*, coding for the Palmitoyl-Protein Thioesterase 1 (PPT1) enzyme, are associated with CLN1 disease (OMIM #256730), a progressive, neurodegenerative disorder of childhood, characterized by acquired microcephaly, hypotonia, delayed psychomotor development followed by deterioration, untreatable seizures and myoclonus, truncal ataxia, and involuntary movements. Children are bedridden by 5–6 years of age, and the fatal outcome occurs by the end of the second decade (Santavuori et al., 1973; Vesa et al., 1995; Mitchison et al., 1998; Simonati et al., 2009). Atypical presentations with an early adult onset have been described (Van Diggelen et al., 2001). The reduced activity of PPT1 is the biomarker of the disease (Hofmann et al., 1997).

PPT1 is a soluble hydrolytic lysosomal enzyme, highly expressed in neuronal cells, which is involved in the degradation of S-acylated proteins by removing the palmitate residues and generating de-palmitoylated proteins suitable for proteasomal degradation and/or recycling. Moreover, it is involved in the regulation of luminal lysosomal acidification (Bagh et al., 2017). In neurons, it is localized in other cellular compartments (such as axons and specialized neural endings) where its enzymatic activity may be conveyed at neutral pH. PPT1 regulates axonal outgrowth as well as dendritic spine number and morphology, thus influencing the pathfinding process and the stabilization of neuronal connectivity (Suopanki et al., 1999; Kang et al., 2008; Holland and Thomas, 2017; Koster and Yoshii, 2019). PPT1 is also associated with several cell functions, including autophagy and mTOR signaling whose regulators are predicted to be palmitoylated proteins (Rebecca et al., 2017).

Palmitoylation, the covalent modification of a protein by S-acylation with palmitate, affects maturation, sorting and trafficking of proteins to specific subcellular compartments (Fukata and Fukata, 2010). Control of palmitoylation occurs via the balance between thioesterases catalyzing the breakdown of the thioester bond, and several enzymes catalyzing its formation. This fine dynamic cycle of palmitoylation/de-palmitoylation is particularly relevant for neurons, since it has been reported that the nervous system alone expresses over 40% of mouse palmitoylome (Sanders et al., 2015). However, despite the availability of animal models KO for *CLN1*, the functional roles as well as the identification of putative substrates of PPT1 in the brain remain somewhat unclear.

In neuronal endings, palmitoylation dynamically regulates the recycling of synaptic vesicles and eventually neurotransmission (Holz and Zimmerberg, 2019; Kreutzberger et al., 2019). Moreover, palmitoylation is a developmentally regulated process affecting plasma membrane remodeling, including the maturation and specification of post-synaptic receptors (Patterson and Skene, 1999; El-Husseini et al., 2002; Tulodziecka et al., 2016). In the post-synaptic microdomain the functional activities of both excitatory and inhibitory receptors are also modulated by palmitoylation (Hayashi et al., 2009; Fukata et al., 2013; Han et al., 2015; Hayashi, 2015; Jeyifous et al., 2016; Itoh et al., 2018). Therefore, it can be assumed that mutated PPT1 affects the turnover of palmitoylated proteins particularly enriched in the synaptic compartment, possibly leading to synaptic dysfunction at both the pre- and postsynaptic regions (Kim et al., 2008; Kielar et al., 2009; Finn et al., 2012; Koster et al., 2019), which may be related to the severe epileptogenicity observed in CLN1 disease.

Multiomic technologies have been utilized in order to depict the molecular network of interacting partners of PPT1, in terms of protein-protein interactions (putative substrates) as well as molecular nodes and pathways, which are affected downstream to PPT1 (Scifo et al., 2015; Tikka et al., 2016; Kline et al., 2019; Sapir et al., 2019). In differentiated SH-SY5Y cells overexpressing *CLN1*, the expression of genes related to the elongation and branching of neuronal structures (including transcripts coding for palmitoylated neuropeptides) was dysregulated. Furthermore, a transcriptional network enclosing several components of the synaptic release machinery and ion channels subunits was identified in the same overexpression system (Pezzini et al., 2017b).

We exploited the observation that a group of genes affected by PPT1 overexpression code for integral membrane proteins belonging to distinct superfamilies of voltage-gated ion channels (VGIC), opening the possibility of correlating transcriptional changes with their functional impact via patch-clamp recordings. At present, no data related to the behavior of VGIC associated with altered expression of *CLN1* are available. Likewise, little is known about the electrical properties and VGIC behavior of SH-SY5Y cells differentiated into a neuronal-like phenotype. By investigating both voltage- gated calcium and potassium channels in differentiated SH-SY5Y cells overexpressing *CLN1*, we aimed to obtain new insights on neuronal excitability and the putative molecular mechanisms as suggested by bioinformatic evaluation and functional annotations. In the long run, the ultimate goal of this study was to contribute to the identification of appropriate molecular targets to alleviate some of the severe clinical manifestations of CLN1 disease, including the drug-resistant epilepsy.

## Materials and Methods

### Cell Lines Culture and Differentiation

SH-SY5Y neuroblastoma cells were cultured in DMEM-High glucose medium supplemented with 15% Fetal Bovine Serum (FBS), 2 mM L-glutamine and 1% non-essential amino acids. Clones overexpressing *CLN1* (SH-*CLN1*) were generated by transfection with pcDNA3 expression vector in which *CLN1* cDNA (NM_000310) was inserted. Cells transfected with the empty vector alone (mock cells; SH-mock) were used as controls. Following antibiotic selection, the isolated clones were then characterized at molecular as well as biochemical level. To induce a more mature neuronal-like phenotype, mock and *CLN1* overexpressing cells were differentiated following a combined approach of retinoic acid and enriched neurobasal medium (RA-NBM). Cells were pre-differentiated for 6 days in RA-containing medium. Next, the medium was changed, and cells were cultured in enriched Neurobasal medium (NBM): this point was considered as day 0 for the electrophysiological study. From day 3, cells were maintained in the same medium and recorded up to day 11 from the start of the differentiation paradigm in enriched NBM. For transcriptomic, morphological as well as biochemical investigations, cells were collected after 3 days in enriched NBM medium [corresponding at day 9 of the original differentiation paradigm previously described in Pezzini et al. (2017a,b)].

### Bioinformatic Analysis of SH-*CLN1* Transcriptomic Profile

The transcriptomic profiles of both mock- and *CLN1-*overexpressing SH-SY5Y cells were previously generated by RNAseq, placed in NCBI's Gene Expression Omnibus and made accessible through GEO Series accession number GSE98834 (Pezzini et al., 2017b). Normalized expression values (calculated as Fragments Per Kilobase per Million mapped reads, FPKM) of each gene was then compared between the two profiles to retrieve differentially expressed genes (DEGs) of SH-*CLN1* cells. The ratio between average FPKM of SH-*CLN1* profile and average FPKM of mock profile was calculated and reported as log_2_ fold change (log_2_FC): transcripts showing a |log_2_(FC)| ≥1 and a False Discovery Rate (FDR, *q*-value) ≤ 0.05 were assigned as DEGs and utilized as input for bioinformatic inquires.

Functional analysis was performed by QIAGEN's Ingenuity® Pathway Analysis (IPA®). Core Analysis was carried out to find most significant functional annotations associated with the differentially expressed genes and to create molecular networks; statistical significance was ascertained by right-tailed Fisher's Exact Test following Benjamini and Hochberg (B-H) correction. Z-score was also evaluated to predict the activation or inhibition of a given biological function. To compare independent Core Analyses, we selected the most meaningful functional annotations (*p* < 0.05 and |z-score|>1) assigned to SH-*CLN1* profile. Free-license databases were also utilized to link the obtained expression profiles to specific cellular processes and/or cellular domains (ToppGeneSuite, https://toppgene.cchmc.org/enrichment.jsp). Cytoscape software (version 3.7.2, http://www.cytoscape.org/) was utilized to draw networks starting from selected GO terms. Lastly, palmitoyl-proteome databases [including, SwissPalm-Protein S-palmitoylation database, http://swisspalm.epfl.ch/; (Blanc et al., 2019)] were inquired to recognize differentially expressed proteins in SH-*CLN1* cells that have been already reported to be modified by palmitoylation.

### Electrophysiology

#### Perforated Patch-clamp Recording

Cells, either mock- or *CLN1*-transfected, were plated on 13 mm diameter round coverslips in 24 well plates and differentiated as described above. Electrophysiological and calcium imaging data have been obtained from cells plated on 33 coverslips during three independent rounds of differentiation. On the day of recording, a coverslip was transferred to a custom made chamber whose bottom was a 0.1 mm-thick coverslip. Cells were visualized using a CCD camera (Leica DFC350 FX) connected to an inverted microscope (Leica DMI 4000) equipped with a 63x objective and controlled by Advanced Fluorescence software (Leica MS). Cells were continuously superfused with Tyrode extracellular saline containing (in mM): NaCl, 130; KCl, 5; CaCl_2_, 2; MgCl_2_, 0.5; glucose, 10; HEPES, 10; pH 7.4 with NaOH. Cells were recorded by the perforated-patch technique using pipettes filled with a 270 μg/ml solution of amphotericin B in pseudo-intracellular saline containing (in mM): KCl 140; NaCl, 10; pipes, 10; pH 7.2 with KOH. Due to the similar intra and extracellular chloride concentrations and sodium and potassium ions mobilities in solution, no corrections were made for junction potentials. Despite the higher series resistance than whole-cell recordings, perforated-patch recording series resistances <80 MΩ proved satisfactory for recording slowly-activating Voltage-gated Potassium Channels (VGKC)-mediated currents from cells with input resistance over 1 GΩ. The use of a voltage-ramp protocol avoided the problems of slow membrane charging when recording fast-activating calcium and barium currents. The VGKC blocker 4-aminopyridine (4-AP) was dissolved in Tyrode at 3 mM final concentration. NS-1643 was prepared as a 6 mM stock solution in DMSO and dissolved to 30 μM final concentration (0.5% DMSO v/v) in Tyrode containing 3 mM 4-AP, with small changes in osmolarity. Blockers were applied via a 1,000 μl pipette tip positioned close to the recorded cell using a mechanical micromanipulator. A six-ways manifold equipped with electrovalves controlled solutions changes.

#### Voltage-Gated Inward and Outward Current Recordings and Analysis

Currents were recorded using an EPC-8 amplifier (HEKA) and filtered by the EPC-8 before sampling by the A/D converter (LIH8+8; HEKA). The LIH8+8 D/A converter also generated the voltage stimuli. Acquisition and voltage stimuli were generated by Patchmaster (HEKA). Inward current through calcium channels were activated by ramping the voltage from−90 to +80 mV in 270 ms, before returning to −90 mV. To reduce noise, 16 sweeps, applied every 5 s, were averaged. The ramp currents were filtered at 1 kHz by a 3-pole Bessel filter before sampling at 5 kHz. When using barium to maximize inward currents through calcium channels, we increased membrane resistance to reduce voltage errors associated with voltage partitioning through the series resistance using extracellular saline containing (in mM): NaCl, 80; KCl, 5; tetraethylammonium (TEA)-chloride 30; BaCl_2_, 20, HEPES, 10; pH 7.4 with NaOH.

Outward currents were activated, from a holding of−80 mV, by 2s-long voltage steps ranging from−70 to +70 mV. Currents were filtered at 0.3 kHz by a 3-pole Bessel filter before sampling at 1 kHz.

Membrane resistance and capacitance assessment: after initial fast capacitance compensation at the time of gigaseal formation, we monitored amphotericin B incorporation by monitoring amplitude and kinetics of capacitive transients. To quantify membrane capacitance, transient capacitive currents generated in response to 45 ms-long 20 mV-voltage steps from a holding potential of−90 mV, were sampled at 50 kHz, filtered at 5 kHz, and numerically integrated using the steady-state current at−70 mV as a baseline to provide the total charge (ΔQ) transferred to the membrane to set the 20 mV voltage change (ΔV). The ratio ΔQ/ΔV then provides the membrane capacitance for each cell recorded. To reduce noise 64 sweeps were averaged. The ratio between ΔV and the net current increase (the difference between currents measured at the end of the 45 ms-long pulse and the holding current at−90 mV) provided the cell membrane resistance. Membrane conductance (G) was computed as the reciprocal of the membrane resistance.

Electrophysiological data were analyzed and plotted using Origin 8.5 (Microcal).

For inward current analysis, we measured net current intensities as the difference between current intensities at the peak of the inward deflection (close to +10 mV) and the current at the same potential of the peak inward deflection extrapolated from the linear fit to the ramp current.

For the analysis of VGKC, we considered both steady-state currents averaged during the last 200 ms of every 2s-long voltage step (200 points) and tail currents. For tail currents analysis, due to their reduction in *CLN1* overexpressing cells for voltages positive to +30 mV, we averaged values over a 20 ms-long interval centered at the peak of the tail current generated by the 2s-long activating step, that in most cells occur at +30 mV. For each 2s-long activating voltage, the fractional activation [FA_(V)_] was computed according to:

(1) $$\begin{array}{l} FA_{\left(V\right)}=\frac{I\left(V\right)-I_{-70}}{I_{\max}-I_{-70}} \end{array}$$

*I(V)* is the tail current measured at a given activating voltage, *I-70* is the tail current measured at−70 mV, and *I*_*max*_ is the maximum tail current. Considering that *I*(*V*) = *G*(*V*)•(*V*−*E*_*K*_) where *G(V)* is the voltage-dependent membrane conductance and *E*_*K*_ is the Nernst potential for potassium ions, *FA*_(*V*)_ values are proportional to the fractional activation of conductance (*GFA*_(*V*)_), and were fitted according to **Equation 2**:

(2) $$\begin{array}{l} GFA_{\left(V\right)}=\frac{1}{1+e^{-\left(\frac{\left(V-V_{0.5}\right)}{S}\right)}} \end{array}$$

*V*_0.5_ is the voltage that activates 50% of the maximum conductance, and *S* is the inverse slope factor that affects the steepness of *GFA*_(*V*)_ activation.

#### Calcium Imaging

A coverslip was washed twice with PBS and then incubated for 25' at room temperature in 5 μM Fluo-4. DMSO concentration was 0.1%. Fluo-4 fluorescence was elicited by the excitation light provided by a 40 nm-wide bandpass filter centered at 470 nm. The emission light, selected by a 50 nm-wide suppression bandpass filter centered at 525 nm, was separated from reflected excitation light through a long-pass dichroic mirror centered at 500 nm. Time-lapse fluorescence was driven by software (Leica AF), using a 200 ms exposure time every 2 s. Voltage-gated calcium channels were activated by membrane depolarization elicited by switching for 30 s the perfusion solution from Tyrode to saline containing (in mM): NaCl, 105; KCl, 30; CaCl_2_, 2; glucose, 10; HEPES 10; pH 7.4 with NaOH. Control recordings indicate membrane depolarizations down to about−40 mV, close to the change of the Nernst potential for potassium.

### Immunoblotting (WB) Analysis

For immunoblotting analysis, 30 μg of RIPA-extracted cellular lysate were separated in polyacrylamide gels, transferred on to PVDF membrane and then probed with primary antibodies over-night at 4°C. Anti-rabbit IgG, HRP-linked F(ab')_2_ fragment antibodies (GE Healthcare) were utilized as secondary antibodies; chemiluminescent detection was performed by Immobilon Western Chemiluminescent HRP Substrate (Merck-Millipore) and images were acquired by UVITEC Q9 Alliance Advanced platform. Densitometric, semi-quantitative analysis was carried out by Fiji software. Primary antibodies used for WB were two rabbit anti-CACNA2D2 antibodies, which recognize either the amino acid residues 850–865 of the protein (1:2000, purchased by Alomone) or the amino acid residues 19-35 (1:2000, purchased by Biorbyt); a rabbit anti-GAPDH polyclonal antibody (1:10.000, Sigma-Aldrich), was used as loading control. To assess the specificity against CACNA2D2 residues 850–865, 1 μg of antibody was incubated with 1 μg of corresponding antigenic peptide supplied by vendor in TBS with 1% no fat dry milk at RT for 1 h; then, the mixture was diluted to reach the appropriate dilution factor in TBS with 0.1% Tween20, 1% not fat dry milk and 0.05% NaN_3_ and the incubation was prolonged over night at 4°C on rotation. In parallel, a mixture with 1 μg of antibody was prepared following the same procedure. Finally, mixtures were used to probe two identical membranes and the immunoblotting analysis was performed as described above.

### De-glycosylation Assay by PNGase F

To remove N-glycosylated residues, 30 μg of protein lysates were precipitated in cold absolute methanol at −20°C for 2 h and centrifuge at 20,000 × g for 30 min at 4°C. Pellets were resuspended in 5 μl of buffer containing 0.5% β-mercaptoethanol and 1% SDS, sonicated and boiled for 5 min. Then, samples were added with 10 μl H_2_O, 1.5 μl 20% CHAPS and with either 3 μl of PNGase F (Sigma-Aldrich) or 3 μl of H_2_O and finally incubated over-night at 37°C. Before loading gels, samples were diluted in 4X Laemli buffer, sonicated, boiled for 5 min and finally resolved by SDS-PAGE under denaturing conditions.

### Immunofluorescence Assay (IF)

Cell grown on coverslips coated by MaxGel (Sigma-Aldrich) were fixed with either cold absolute methanol (for KNCH4 IF) or with 4% paraformaldehyde (for CACNA2D2 IF). Coverslips were then blocked with Normal Goat serum and incubated with primary antibody over-night at 4°C. Primary antibodies used for IF were two rabbit polyclonal antibodies, anti-CACNA2D2 (1:500) and anti-KCNH4 (1:50; all purchased by Biorbyt). To detect bound primary antibody, host species-related secondary antibodies conjugated either with AlexaFluor 488 or 594 dyes (Molecular Probes, Thermo Fisher) were used whereas nuclei were counterstained with 5 μg/ml DAPI (4',6-diamidino-2-phenylindole dihydrochloride, Sigma-Aldrich). Images were acquired by an AxioLab microscope (equipped with an AxioCam and AxioVision 4.3 software, Zeiss). In addition, confocal microscopy analysis was performed on a LEICA TCS-SP5 inverted confocal microscope using a high NA oil immersion 40x objective; images were acquired by LasX software and further analyzed by Fiji. For quantitative evaluation, cells were selected by ROI and assessed in all z-planes. The number of CACNA2D2 immunolabelled dot structures were manually counted and tracked in all planes. To evaluate fluorescent intensity of KCNH4, corrected total cell fluorescence (CTCF) were calculated as follows: Integrated Density—(ROI area X mean gray value fluorescence of background).

### Statistical Analysis

For the electrophysiological study, we preliminarily assessed the deviation of data from a normal distribution by the Shapiro-Wilk test. In case of deviation, two samples comparisons were carried out by the non-parametric Mann-Whitney *U*-test. We used ANCOVA to evaluate the impact of categorical variables, such as genotype and charge carrier, and continuous variables such as membrane capacitance or days after completing differentiation. In case of multiple drug applications to the same cell, two-way ANOVA with repeated measurements in time evaluated the significance of the observe effects. In case of significant effects, *post-hoc* comparisons were carried out by t-tests using the residual variance generated by either ANCOVA or ANOVA and applying the Bonferroni's correction for multiple comparisons.

For biochemical and morphological quantitative evaluations, statistical analyses and representation of data were carried out by Prism 8 (GraphPad Software, San Diego CA). For WB, protein isolation was performed at least in three independent experiments of neuronal differentiation; densitometric data were presented as mean ± standard deviation (*SD*) and statistical significance was analyzed by two-way ANOVA. For immunofluorescence analysis, we compared mock- and *CLN1* transfected cells by either two-tailed unpaired *t*-test or by Mann-Whitney *U*-test following ascertainment of normal distribution of data by Shapiro-Wilk normality test.

## Results

### Bioinformatic Investigation of the Transcriptome of *CLN1* Overexpressing Cells: Focus on Voltage-Gated Ion Channels

Bioinformatic data of the transcriptome of RA-NBM differentiated SH-*CLN1* cells revealed a meaningful association of their transcriptomic profile with GO terms related to synaptic functioning and ion channel activity (Supplementary Figures 1, 2). We focused on DEGs coding for subunits of Voltage-gated Calcium Channels (VGCC) and Voltage-gated Potassium Channels (VGKC or K_v_), which play relevant role in neuronal excitability (Table 1).

**Table 1:** Differentially expressed genes (DEGs) of SH-*CLN1* transcriptomic profile coding for subunits of voltage-gated calcium channels (VGCC) and voltage-gated potassium channels (VGKC).

**Family**	**Gene**	**Alias**	**Gene Name**	**SH-Mock**	**SH-*****CLN1***		
				**FPKM**	**FPKM**	**log_**2**_FC**	***q*-value**
**Voltage-gated Calcium Channels (VGCC)**							
Voltage-gated calcium channel auxiliary α2δ subunits	*CACNA2D2*	α2δ-2	Calcium voltage-gated channel auxiliary subunit alpha2delta 2	132.30	61.13	**−1.11**	0.001
	*CACNA2D3*	α2δ-3	Calcium voltage-gated channel auxiliary subunit alpha2delta 3	1.86	7.11	**1.94**	0.001
Calcium channel auxiliary γ subunits	*CACNG2*	*γ2*	Calcium voltage-gated channel auxiliary subunit gamma 2	3.13	1.46	**−1.10**	0.037
**Potassium Channels**							
Voltage-gated potassium channels (K_v_)	*KCNA3*	K_v_1.3	Potassium voltage-gated channel subfamily A member 3	1.26	0.26	**−2.25**	0.002
	*KCNB1*	K_v_2.1	Potassium voltage-gated channel subfamily B member 1	2.78	1.23	**−1.18**	0.001
	*KCNH4*	K_v_12.3	Potassium voltage-gated channel subfamily H member 4	1.50	0.67	**−1.16**	0.006
	*KCNH6*	K_v_11.2	Potassium voltage-gated channel subfamily H member 6	16.04	7.82	**−1.04**	0.001
	*KCNQ3*	K_v_7.3	Potassium voltage-gated channel subfamily Q member 3	0.93	0.17	**−2.46**	0.001
	*KCNQ5*	K_v_7.5	Potassium voltage-gated channel subfamily Q member 5	1.95	4.46	**1.20**	0.029
Potassium two pore domain channels	*KCNK1*	K_2P_1.1	Potassium two pore domain channel subfamily K member 1	19.93	7.27	**−1.46**	0.001
	*KCNK3*	K_2P_3.1	Potassium two pore domain channel subfamily K member 3	6.84	3.13	**−1.13**	0.001
	*KCNK6*	K_2P_6.1	Potassium two pore domain channel subfamily K member 6	1.82	0.86	**−1.09**	0.015
Potassium inwardly rectifying channels	*KCNJ2*	K_ir_2.1	Potassium inwardly rectifying channel subfamily J member 2	2.14	0.71	**−1.59**	0.003

DEGs of SH-CLN1 transcriptomic profile, coding for either Calcium or Potassium channel subunits, are reported (|log_2_FC| > 1, q-value < 0.05). For a wider compendium of VGCC and Potassium channels encoding genes expressed in mock and SH-CLN1 cell lines, see Supplementary Materials and Supplementary Tables 1, 2.

*Family, Channel Gene Family; Gene, NCBI Gene Symbol; Alias, Alternative name (including symbol according NC-IUPHAR -Nomenclature Committee of the International Union of Pharmacology); Gene Name, HGNC Approved Name; FPKM, fragments per kilobase of exon per million fragments mapped; log_2_FC, log_2_ fold change (SH-CLN1 vs. SH-mock); q-value, false discovery rate (FDR)*.

In reference to VGCC, *CLN1* overexpression affects the expression of two genes, namely *CACNA2D2* and *CACNA2D3*, coding for two different α_2_δ subunits, which were found to be down- and up-regulated, respectively. It is of note that these two DEGs belong to the subfamily of Auxiliary Alpha2Delta Subunits, which enclose four genes only (three expressed in our cell system; Supplementary Table 1). No significant changes in the expression of genes encoding other VGCC subunits were observed (Supplementary Table 1).

According to GO categories 10 DEGs of SH-*CLN1* transcriptome code for different potassium channels and 6 of them belong to VGKC (K_v_) subfamily. Taking into account the total number of K_v_ genes expressed in mock and SH-*CLN1* cells (*n* = 22), the percentage of dysregulated K_v_ DEGs in *CLN1* overexpressing cells was about one-third (Supplementary Table 2).

### Electrophysiological Properties of Differentiated Mock and SH-*CLN1* Cells

**Figure 1:**
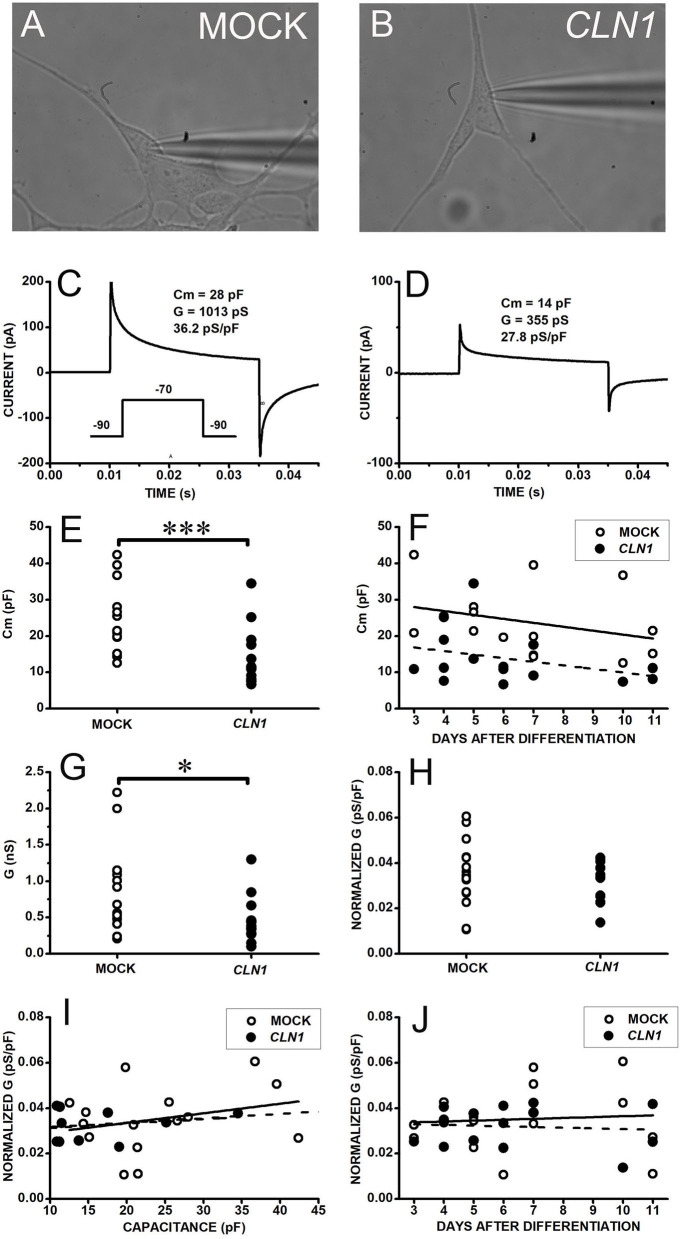
**(A,B)** Bright field pictures of differentiated SH-SY5Y cells, either mock- **(A)** or *CLN1*-transfected **(B)**, during perforated patch-clamp recording. **(C,D)** Response of cells in **(A,B)** to 45 ms-long voltage steps from−90 to−70 mV for assessment of membrane resistance and capacitance. The transient currents are larger in the mock-transfected **(C)** than in wt*CLN*1-transfected cell **(D)**, corresponding to membrane capacitance (Cm) of 28 and 14 pF, respectively. **(E)** Membrane capacitance is significantly higher in mock- (open circles) as compared to *CLN1*-transfected (filled circles) cells (***P < 0.001, Mann-Whitney *U*-test). **(F)** Symbols plot Cm values for mock (open circles) and *CLN1*-transfected (filled circles) cells as a function of days after completing the differentiation protocol. Straight lines plot best fit to mock- (tick continuous line) and *CLN1*-transfected (dashed line). One-way ANCOVA found a significant effect of genotype [*F*_(1,27)_ = 12.81, *P* = 0.0013], but not of days after differentiation [*F*_(1,26)_ = 0.0072, *P* = 0.921]. **(G)** Datapoints plot membrane conductance values (see section voltage-gated inward and outward current recordings and analysis in Materials and Methods) of mock (open symbols) and *CLN1*-transfected (filled symbols) cells. *P < 0.05 by two-tailed independent *t*-test (*t* = 2.497 with 28 df, *P* = 0.0187). **(H)** Membrane conductance values normalized to membrane capacitance (pS/pF) of mock (open circles) and *CLN1*-transfected (filled symbols) cells. Average values di not differ significantly (*t* = 0.72804 with 28 df, *P* = 0.473 by independent *t*-test). **(I)** Datapoints plot normalized conductance data from mock- (open circles) and *CLN1*-transfected (filled circles) cells as a function of membrane capacitance. Best-fitting lines had similar slopes of 0.422 and 0.195 pS/pF for mock- (thick continuous) and *CLN1*-transfected (dashed line) cells. One-way ANCOVA indicate lack of significant effects by both genotype [*F*_(1,27)_ = 0.24, *P* = 1.0000] and Cm [*F*_(1,26)_ = 0.17, *P* = 0.6835]. **(J)** Datapoints plot normalized conductance values as a function of days after differentiation for mock (open circles) and *CLN1*-transfected (filled circles) cells. Straight lines plot best fit mock- (thick) and *CLN1*-transfected (dashed). One-way ANCOVA indicates both genotype [*F*_(1,27)_ = 0.5, *P* = 0.4856] and days after differentiation [*F*_(1,26)_ = 0.15, *P* = 0.7017] do not reach significance.

We selected the perforated-patch technique to assess the functional impact of the dysregulated expression of genes coding for VGIC upon *CLN1* overexpression. This approach minimizes intracellular milieu changes that may affect VGIC during long recordings (longer than 30 min) required to evaluate both VGCC and VGKC through the serial applications of blockers/modulators. We initially evaluated the possible occurrence of unspecific effects in response to *CLN1* overexpression. Specifically, we compared the basic electrophysiological properties of differentiated SH-SY5Y cells (see methods), either mock-transfected or overexpressing *CLN1*. Data in Figure 1 plot currents evoked in response to a 45 ms-long voltage pulse from−90 to−70 mV to estimate cell membrane capacitance and resistance. Both mock- (Figure 1A) and *CLN1*- (Figure 1B) transfected cells had similar 0-current levels (a proxy for the resting membrane potential) close to−90 mV. The average (±SEM) holding currents at−90 mV were−0.38 ± 0.15 and−0.35 ± 0.16 pA/pF for mock and *CLN1* cells, consistent with the notion of similar membrane potentials for mock and *CLN1*-overexpressing cells. The mock cell membrane capacitance of 28 pF (Figure 1C), twice the 14 pF of the *CLN1*-transfected cell (Figure 1D), indicates a large reduction in the plasma membrane surface in response to *CLN1* overexpression. Data in panel E depict the distributions of membrane capacitances (Cm) measured in 15 mock- and 15 *CLN1*-transfected cells, indicating the significant reduction in the cell surface induced by *CLN1* overexpression (*P* = 0.0007 by Mann-Whitney *U*-test). In Figure 1F, linear regression fits of membrane capacitance as a function of days after differentiation indicate similar slopes (−1.08 and −0.98 pF/day for mock- and *CLN1*-transfected cells, respectively). Analysis by one-way ANCOVA for the effects of genotype and time after differentiation confirmed a significant effect of genotype [*F*_(1,27)_ = 12.81, *P* = 0.0013], while the effect of days elapsed after completing the differentiation protocol did not reach significance [*F*_(1,26)_ = 0.007, *P* = 0.9211). Consistent with their larger membrane capacitance, mock cells also had significantly higher membrane conductance than *CLN1* cells (*t* = 2.497 with 28 df, *P* = 0.0187 by two-sided independent *t*-test, Figure 1G). Despite the large difference in plasma membrane area, average (±SEM) normalized conductance of 35.13 ± 3.78 and 31.93 ± 2.26 pS/pF for mock- and *CLN1*-transfected cells, respectively, did not differ significantly (*t* = 0.72804 with 28 df, *P* = 0.473 by independent *t*-test) suggesting that increased levels of *CLN1* do not lead to unspecific cell damage (Figure 1H). We also tested the impact on normalized conductance of membrane capacitance, an index of cell size, and days after differentiation. As shown in panel I, plotting normalized conductance data from mock- and *CLN1*-transfected cells as a function of membrane capacitance, best-fitting lines had similar slopes (0.422 and 0.195 pS/pF for mock-and *CLN1*-transfected cells, respectively). One-way ANCOVA indicates that neither genotype [*F*_(1,27)_ = 0.24, *P* = 1.0000], nor membrane capacitance [*F*_(1,26)_ = 0.17 *P* = 0.6835] had significant effects, suggesting that the number of channels for plasma membrane unit area is largely unaffected by differences in cell size, irrespective of *CLN1* overexpression. Data in panel J, plotting normalized conductance values as a function of days after differentiation, were interpolated by best-fitting straight lines with slopes of 0.387 and−0.302 pS/pF for mock- and *CLN1*-transfected cells. One-way ANCOVA indicates that neither genotype [*F*_(1,27)_ = 0.5, *P* = 0.4856], nor days after differentiation [*F*_(1,26)_ = 0.15, *P* = 0.7017] had significant effects, suggesting that the number of membrane channels for plasma membrane unit area is largely unaffected by the time elapsed after differentiation, irrespective of *CLN1* overexpression.

### Voltage-Gated Calcium Channel

#### Electrophysiological Investigations and Intracellular Ca^2+^ Flux

To assess the functional relevance of the dysregulated expression of genes coding for VGCC, we compared calcium currents in differentiated SH-SY5Y cells, both SH-mock and SH-*CLN1* cell lines. Transcriptomic data do not show a significant change of specific pore-forming subunits. We then opted for a quick evaluation of inward currents using a voltage-ramp protocol, rather than adopting the usual voltage step protocol used to assess the kinetics and the voltage dependence of VGCC and resolve the contribution of specific isoforms.

**Figure 2:**
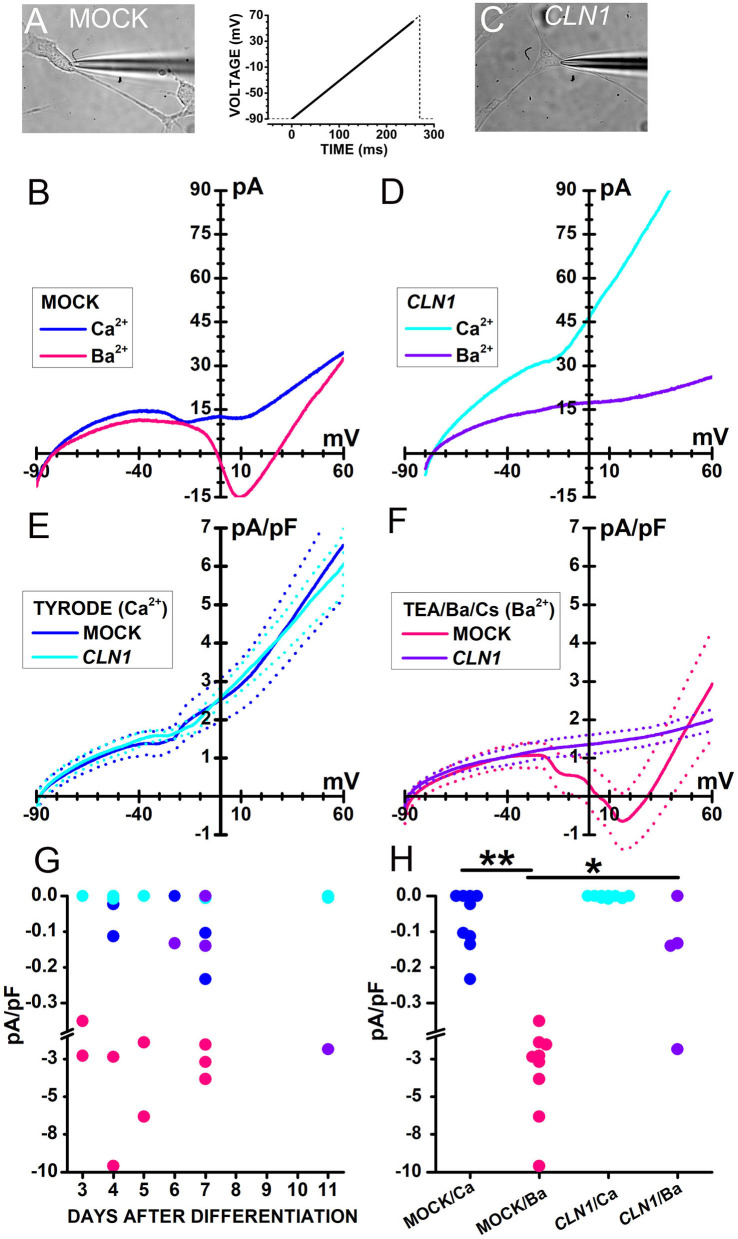
**(A,C)** Bright-field images of a mock-transfected **(A)** and a *CLN1*-transfected **(C)** cell during perforated-patch recording by a patch pipette. **(B)** Traces plot representative currents, recorded from the cell in **(A)**, in response to a voltage ramp [see the thick trace in the inset between **(A)** and **(C)**] in either Tyrode (blue traces) or Ba^2+^/TEA solutions (magenta). **(D)** Traces plot representative currents, recorded from the cell in **(C)**, in response to a voltage ramp [see inset between **(A)** and **(C)**] either in Tyrode (cyan) or in Ba^2+^/TEA solutions (purple). In **(B,D)**, each trace is the average of 16 sweeps, recorded at 5s-intervals. **(E)** Continuous and dashed traces plot average ramp normalized currents (pA/pF) and their standard error, respectively, from mock- (blue, *n* = 9) and *CLN1*-transfected (cyan, *n* = 9) cells in Tyrode. **(F)** Continuous and dashed traces plot average ramp currents and their standard error, respectively, from mock- (pink, *n* = 9) and *CLN1*-transfected (purple, *n* = 4) cells in Ba^2+^/TEA solution. **(G)** Symbols plot normalized inward current amplitudes for mock cells in either Ca^2+^-containing (blue) or Ba^2+^-containing (pink) saline and *CLN1*-transfected cells in either Ca^2+^-containing (cyan) or Ba^2+^-containing (purple) saline. Note axis break from−0,38 to−1,0 pA/pF. Two-way ANCOVA for the effects of genotype and charge carrier and days after differentiation covariant indicates significant effects of both genotype [*F*_(1,26)_ = 6.05, *P* = 0.0209] and charge carrier [*F*_(1,26)_ = 17.34, *P* = 0.0003], with no significant effects for their interaction [*F*_(1,26)_ = 1.45, *P* = 0.2394] as well as for the time after differentiation [*F*_(3,23)_ = 0.23, *P* = 0.8745]. **(H)** *post-hoc* comparisons by *t*-test using Bonferroni's correction found significant differences between currents recorded from mock-transfected cells using either Ba^2+^ or Ca^2+^ as charge carrier (**t = 4.314 with 16 df, *P* < 0.01) and between currents recorded from mock- and *CLN1*-transfected cells using Ba^2+^ as charge carrier (*t = 3.012 with 11 df, *P* < 0.05). Note axis break from−0,38 to−1,0 pA/pF.

Sweeps in Figure 2 plot currents evoked in response to a voltage ramp (see inset between A and C) in representative cells either mock- (B) or *CLN1*-transfected (D) cells recorded in Tyrode's solution (blue and cyan traces in B and D, respectively) containing 2 mM CaCl_2_. In the range from−20 to−10 mV, small inward humps are present in both cells, but a second inward deflection between 0 and +10 mV is present in the mock cell only. To evaluate whether these small inward currents represent genuine Ca^2+^ currents, we exploited the higher permeability of Ba^2+^ through VGCC. In the presence of 20 mM Ba^2+^ as charge carrier, a larger inward current, peaking close to 10 mV, flows in the representative mock cell than in Tyrode (pink and blue sweeps in Figure 2B, respectively). Ba^2+^ does not lead to a large inward current increase in the representative *CLN1* overexpressing cell (purple sweep in Figure 2D).

Data in panels 2E and 2F illustrate the difference between mock and *CLN1*-transfected cells when using Ba^2+^ as charge carrier. Average currents normalized to membrane capacitance from 9 mock- and 9 *CLN1*-transfected cells (Figure 2E, thick blue, and cyan traces, respectively) showed similar amplitudes at every voltage when recorded in Tyrode. On the other hand, a remarkable difference was observed for currents recorded in the presence of 20 mM Ba^2+^ from 9 mock- and 4 *CLN1*-transfected cells (Figure 2F, thick continuous pink and purple traces, respectively), with a large inward current peaking at about +10 mV in the mock-transfected cells only. Symbols in Figure 2G plot peak inward currents measured in mock (blue and pink) and *CLN1* overexpressing (cyan and purple) cells, either in 2 mM Ca^2+^ (Tyrode, blue and cyan circles) or 20 mM Ba^2+^ (TEA/Ba/Cs, pink and purple symbols) as a function of days after differentiation. A 2-way ANCOVA found significant effects of both genotype [*F*_(1,26)_ = 6.05, *P* = 0.0209] and charge carrier [*F*_(1,26)_ = 17.34, *P* = 0.0003], with no significant effects for their interaction [*F*_(1,26)_ = 1.45, *P* = 0.2394] as well as for the time after differentiation [*F*_(3, 23)_ = 0.23, *P* = 0.8745]. As shown in Figure 2H, *post-hoc t*-tests found a significant difference for charge carrier in mock cells (^**^t = 4.314 with 16 df, *P* < 0.01) and between mock- and *CLN1*-transfected cells in Ba^2+^ (^*^t = 3.012 with 16 df, *P* < 0.05). A similar analysis on the impact of membrane capacitance (Cm) on the effects of genotype and charge carriers on inward current amplitude (not shown) indicates a significant effect of charge carrier [*F*_(1,26)_ = 16.8, *P* = 0.0004], but neither genotype [*F*_(1,26)_ = 2.71, *P* = 0.1118], nor the interaction charge carrier/genotype [*F*_(1,26)_ = 1.95, *P* = 0.1744] had significant effects.

Considering that we observed differences in inward currents between mock- and *CLN1*-transfected cells in the presence of high concentrations of external Ba^2+^, we used calcium imaging to provide an independent confirmation of *CLN1* impact on VGCC in the presence of physiological levels of external Ca^2+^.

**Figure 3:**
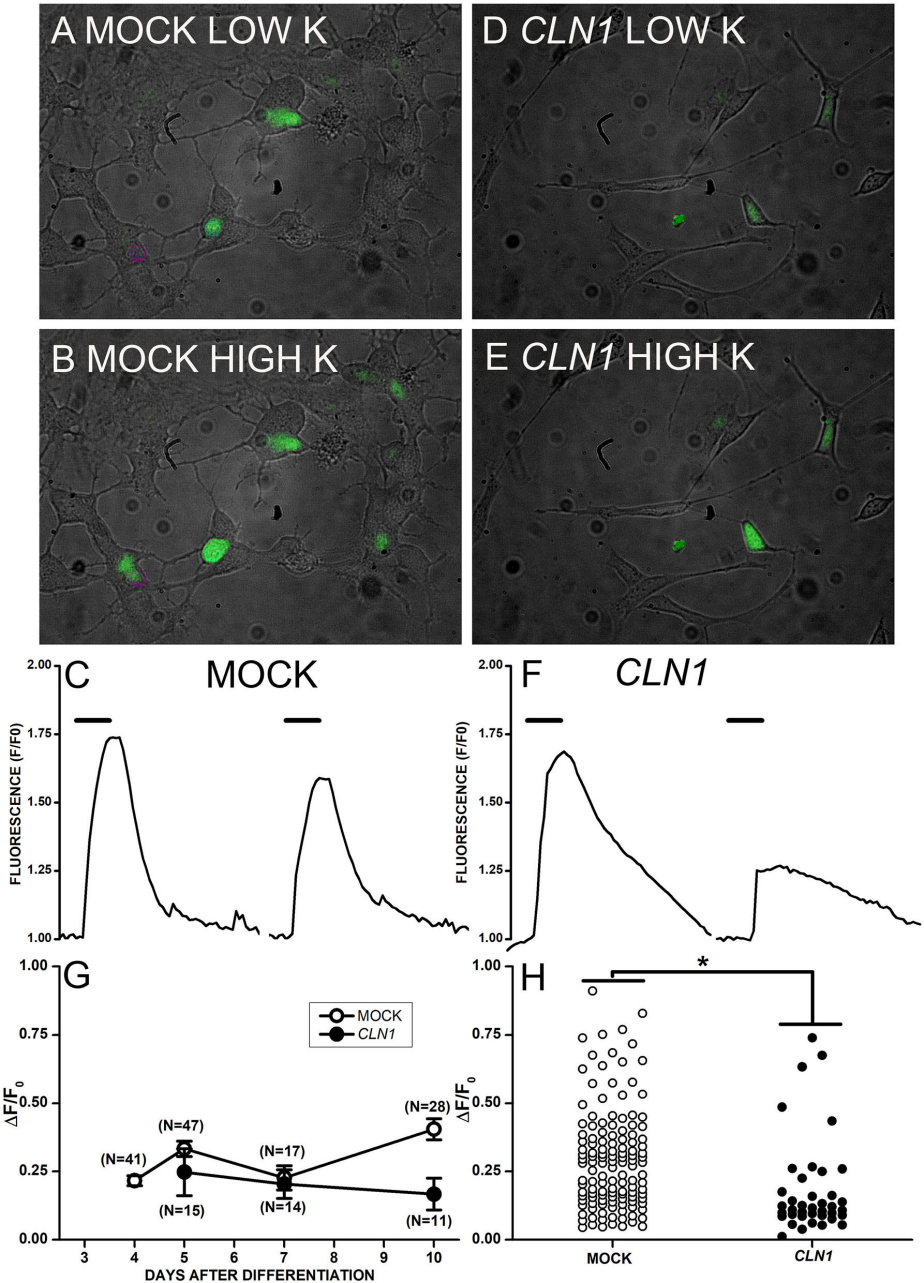
**(A,B,D,E)** Superimposed bright-field and fluorescent images collected from mock- **(A,B)** and *CLN1*-transfected **(D,E)** cells in Tyrode **(A,D)** and during application of modified Tyrode with 30 mM KCl **(B,E)**. **(C,F)** Time course of fluorescence normalized to the pre-KCl application (F_0_) for two mock- **(C)** and two *CLN1*-transfected **(F)** cells from pictures in **(B,E)**, respectively. Thick segments above the experimental traces plot the time of application of saline with 30 mM KCl. **(G)** Datapoints plot average ΔF/F_0_ values ±1 SEM for mock (open symbols) and *CLN1*-transfected (filled symbols) cells as a function of days after differentiation. One-way ANCOVA indicates a non-significant effect for time after differentiation [*F*_(1,169)_ = 2.63, *P* = 0.05]. **(H)** Plot of changes in normalized fluorescence values (ΔF/F_0_) values for 132 mock- (open circles) and 40 *CLN1*-transfected (filled circles) cells. The fluorescence increase is significantly higher in mock- than in wt*CLN1*-transfected cells (*P < 0.05) by one-way ANCOVA, indicating a significant effect for genotype [*F*_(1,170)_ = 5.97, *P* = 0.0156).

The application of saline containing 30 mM KCl to mock-transfected cells in Figure 3A generated a large increase in Fluo-4 fluorescence (Figure 3B). On the other hand, 30 mM KCl induced fluorescence increase in *CLN1*-transfected cells (Figures 3D,E) of reduced intensity as compared to mock cells. The time-course of fluorescence (F) normalized to basal levels (F_0_) for two mock and two CLN1 overexpressing cells (see panels A, B, and D, E above) is plotted in Figure 3C and 3F, respectively. Note that the kinetics of fluorescence decay in both mock and *CLN1* overexpressing cells fall within the range observed in both genotypes. Mean values (± 1 SEM) of the increase in fluorescence normalized to basal fluorescence (ΔF/F_0_) measured in mock- (empty circles) and *CLN1*-transfected (filled circles) cells are plotted as a function of days after differentiation in Figure 3G. A one-way ANCOVA indicates a significant effect of genotype [*F*_(1,170)_ = 5.97, *P* = 0.0156], while the effect of time after differentiation was not significant [*F*_(1,169)_ = 2.63, *P* = 0.1067]. Dot-plot in Figure 3H shows that a large fluorescence increase may occur in both mock- and *CLN1*-transfected cells, but the significant difference (^*^P = 0.0156 by ANCOVA) provides independent support to the notion that *CLN1*-transfection reduces the number of functional VGCC.

### Expression of the VGCC Subunit CACNA2D2

**Figure 4:**
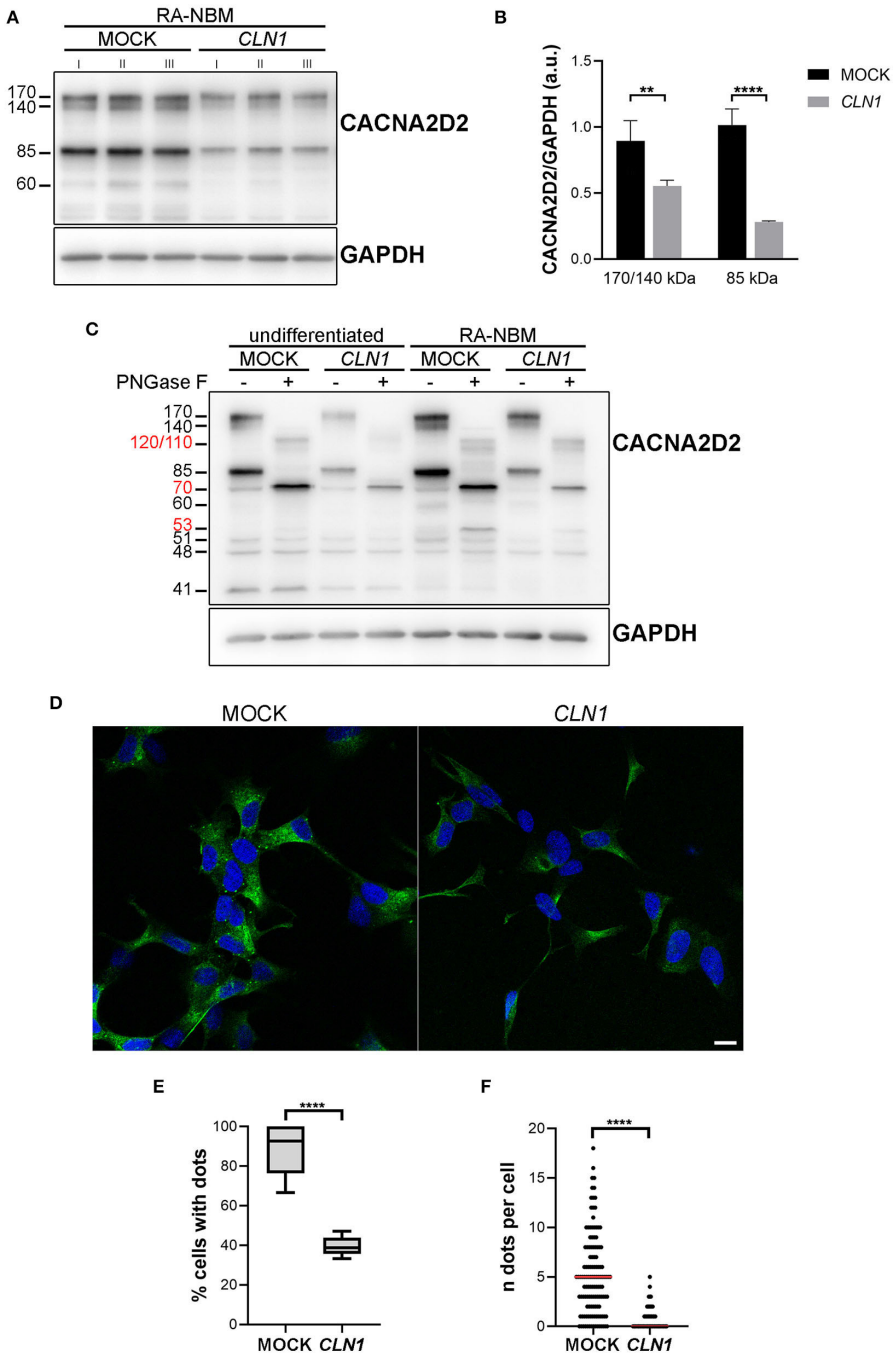
Biochemical and morphological investigations of CACNA2D2 expression on mock and *CLN1*-transfected cell lines. **(A)** Immunoblot analysis of CACNA2D2 expression on homogenates of mock- and *CLN1*-transfected cells, cultured in RA-NBM differentiating medium. A higher expression of both 170/140 and 85 kDa bands was evident in lysates from mock cells as compared to lysates from *CLN1*-transfected cells. **(B)** Densitometric evaluation of 170/140 and 85 kDa isoforms, which putatively represents two glycosylated isoforms, confirmed a significant reduced expression in differentiated *CLN1*-transfected cells compared to mock. Two-way ANOVA followed by Bonferroni multiple comparisons test; **P < 0.01, ****P < 0.0001; I, II, and III represent cellular homogenates from three independent differentiation experiments. **(C)** Treatment with PNGase F induced a shift of 170/140 and 85 kDa bands, generating new bands running ~at 120/110 and ~70 kDa, respectively (see molecular weight in red). Another band of 60 kDa, present only in the differentiated cells, disappeared following PNGase F treatment and likely generated a 53 kDa immunoreactive band. A quantitative difference in the expression of 170/140 and 85 kDa bands was also evident between mock and *CLN1-* transfected cells, both under undifferentiated conditions and following RA-NBM differentiation. **(D)** Immunofluorescence assay using the rabbit polyclonal antibody recognizing the extracellular N-term of the protein showed a clear dot-like pattern on mock cells, mainly localized on the cell body; on *CLN1*-transfected cells, the pattern of staining was more diffuse and less cells with immunolabelled dots were observed. The assay was performed without the permeabilization step in order to visualize the protein exposed on the cell membrane. Scale bar equals to 10 μm. **(E)** The quantitative analysis of immunolabelled cells showed a higher percentage of mock cells showing at least one dot, as compared to *CLN1-*transfected cells. ****P < 0.0001, unpaired *t*-test. **(F)** In addition, the number of detected immunolabelled dots was lower in *CLN1-*transfected cells (*N* = 82) as compared to mock cells (*N* = 127). ****P < 0.0001, Mann-Whitney *U*-test.

We subsequently investigated whether the downregulation of *CACNA2D2* in *CLN1* overexpressing cells led to a reduction of the expression of the corresponding protein CACNA2D2 (also known as α2δ-2), a subunit of VGGC necessary for their appropriate functioning. We utilized the rabbit polyclonal antibody which recognizes the amino acids residues 850–865. Semi-quantitative immunoblotting analysis of lysates extracted from differentiated *CLN1*-transfected cells pinpointed a significant reduction of the 170/140 and 85 kDa bands as compared to mock lysates, in agreement with *CACNA2D2* downregulation (Figures 4A,B). A de-glycosylation assay confirmed that the 170/140 and 85 kDa bands corresponded to glycosylated isoforms of CACNA2D2, since the PNGase F treatment induced a definite shift in the molecular weight of these two bands (Figure 4C). Moreover, a modulation in their expression was also evident under different culture conditions, in particular an upregulation was evident following RA-NBM differentiation for both cell lines, especially in mock cells. Pre-incubation of the antibody with the corresponding antigenic peptide abolished the immunoreactivity, in accordance with the specific binding of this antibody (Supplementary Figure 3). Similar WB findings (namely, immunoreactive bands and signal modulation between mock and *CLN1*-trasfected cells as well as between undifferentiated and differentiated conditions) were detected using another polyclonal antibody (Supplementary Figure 4). However, in this case it was not possible to test the specificity of the antibody with the corresponding antigenic peptide.

Immunofluorescence assay revealed a bright punctate pattern in mock cells, mainly in the cell bodies, whereas few dots were detected in SH-*CLN1* cells (Figure 4D). Quantitative evaluation of immunolabelled cells showed a significant reduction in labeling in *CLN1*-transfected cells as compared to mock ones (Figures 4E,F).

### Voltage-Gated Potassium Channel

#### Electrophysiological Investigations

**Figure 5:**
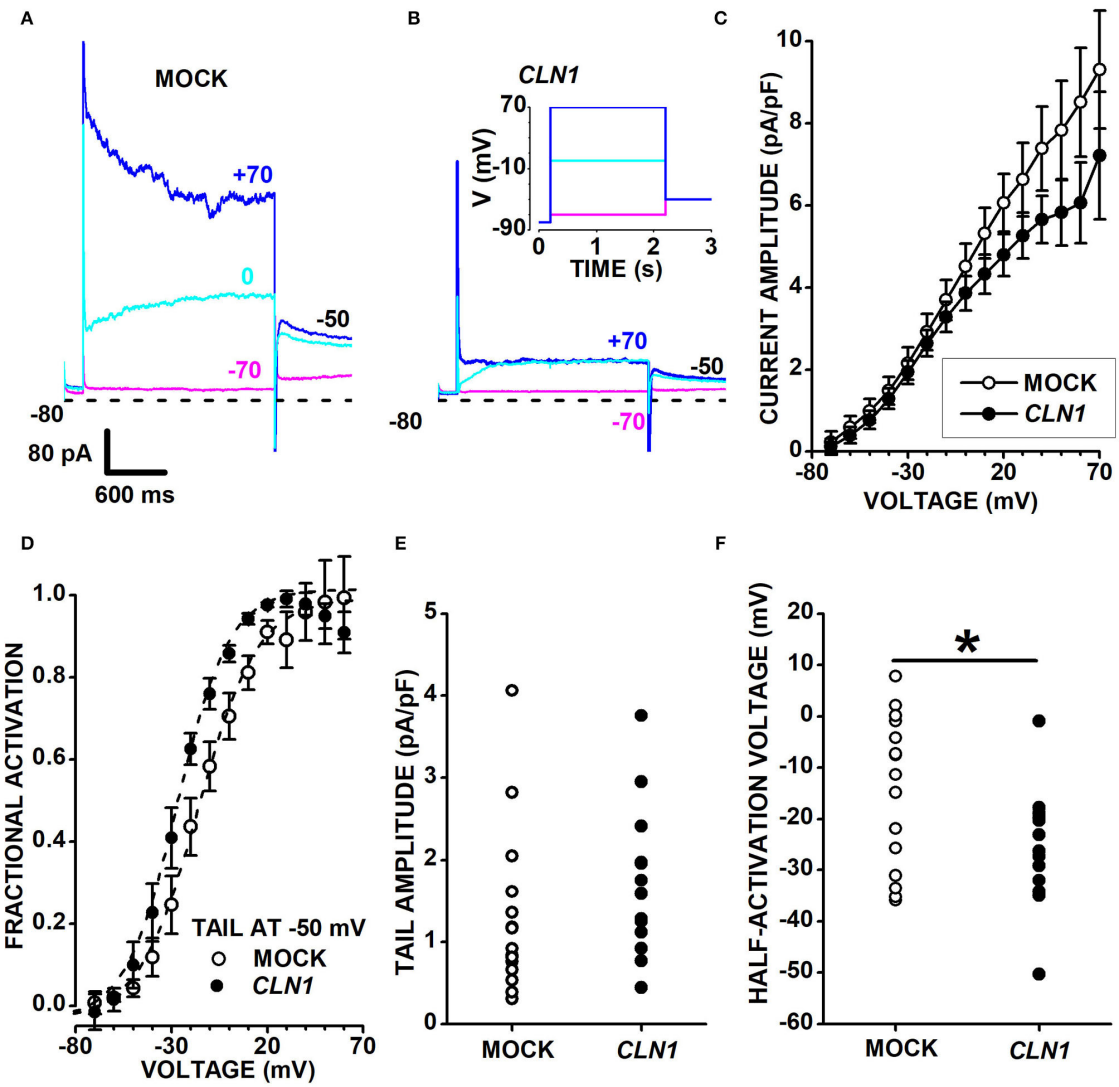
**(A,B)** Sweeps plot currents recorded from a mock- **(A)** and a *CLN1*-transfected **(B)** cell, in response to 2s-long voltage steps at−70 (magenta), 0 (cyan), and +70 mV (blue), from a holding of −80 mV. Tail currents are recorded at −50 mV. The inset in **(B)** plots the voltage stimulation protocol. In **(A,B)**, short dash lines indicate the 0-current level. **(C)** Average currents from 15 mock- (open circles) and 15 *CLN1*-transfected (filled circles) cells with their sem. **(D)** Data points plot the average values ± 1 SEM of tail currents fractional activation at −50 mV (see Materials and Methods) as a function of the voltage applied during the 2s-long voltage step preceding the −50 mV voltage step. Dashed lines plot best fit of **Equation 2** to mock (open circles) and *CLN1*-transfected (filled circles) data points. Average half-activation voltages of mock (−14.6 ± 3.8 mV, *N* = 15) and *CLN1*-transfected (−25.8 ± 3.2 mV, *N* = 13) cells were significantly different (*t* = 2.187 with 26 df, *P* = 0.0379) by two-tailed independent *t*-test. **(E)** Data points plot tail current amplitudes for mock (open circles) and *CLN1*-transfected (filled circles) (*t* = 1.104 with 26 df, *P* = 0.2799 by independent *t*-test). **(F)** Data points plot tail currents half-activation voltages for mock (open circles) and *CLN1*-transfected (filled circles) data. One-way ANCOVA indicates a significant effect of genotype [*F_(1,25)_ = 6.39, *P* = 0.0182], but time after differentiation did not significantly affect the half-activation voltages [*F*_(1,24)_ = 0.01, *P* = 0.9212].

*CLN1* transfection significantly reduces the expression of KCNH isoforms subfamily (K_v_11 and K_v_12) as well as of Kv7 channels of the KCNQ subfamily of VGKC. These isoforms are expressed by Central and Peripheral Nervous System neurons (Zou et al., 2003) and carry slowly-activating and deactivating currents. Sweeps in Figure 5 plot representative currents activated by 2s-long voltage pulses at−80, 0, and +70 mV (see inset in panel B), applied from a holding voltage of−80 mV, and show slowly-activating currents at 0 mV in both the mock and *CLN1*-transfected cells (cyan sweeps in Figures 5A,B, respectively). Despite the difference in current amplitudes of representative mock and *CLN1* cells in 5A and 5B, average normalized currents measured at the end of the 2s-long voltage step nearly superimpose up to−10 mV, diverging for voltage positive to−10 mV, although with substantial SEM (Figure 5C). To assess whether genotype affects VGKC, outward currents normalized to membrane conductance were converted into conductances and fitted by Boltzmann equation (see **Equation 2** in the Methods section). Data in Supplementary Figure 5A plot average normalized conductance with their SEM for mock (open circles) and *CLN1*-transfected (filled circles) cells, along with best fitting Boltzmann equations. Panel SF5B plots the average inverse slope factors (S) and show that *CLN1*-transfected cells (11.92 ± 1.06 mV, *N* = 14) has a significantly steeper activation than mock cells (17.77 ± 2.36 mV, *N* = 15) (*t* = 2.206 with 27 df, *P* = 0.036 by independent *t*-test). Average maximal normalized conductances of mock (67.89 ± 17.59 pS/pF) and *CLN1*-transfected cells (39.81 ± 5.58 pS/pF) in Supplementary Figure 5C did not differ significantly (*t* = 1.478 with 27 df, *P* = 0.151 by two-tailed independent *t*-test). Also, the average half-activation voltages of mock (-23.87 ± 7.07 mV) and *CLN1*-transfected cells (-35.79 ± 3.96 mV) in Supplementary Figure 5D did not differ significantly (*t* = 1.441 with 27 df, *P* = 0.161). We also evaluated by one-way ANCOVA whether days after differentiation contribute a quote of variance that may blur the significant difference between genotypes in either maximal conductance or half-activation voltage. However, ANCOVA indicates a lack of significant effect for both genotype and days after differentiation on maximal conductance [genotype *F*_(1,26)_ = 2.26, *P* = 0.1448; days after differentiation *F*_(1,25)_ = 2.63, *P* = 0.1174] and half-activation voltage [genotype *F*_(1,26)_ = 1.95, *P* = 0.1744; days after differentiation *F*_(1,25)_ = 0.03, *P* = 0.8639].

We considered that several genes in differentiated SH-SY5Y cells, such as *KCNH2, KCNH6, KCNH4, KCNH8*, and *KCNQ2*, code for VGKC carrying outward currents. In these conditions, the two-fold reduction of *KCNH4* (K_v_12.3), a low-expressed DEG, and the slight downregulation of *KCNH8* (K_v_12.1; see Supplementary Table 2) may hardly translate into a significant decrease in the amplitude of outward currents due to the presence of K_v_11 and other K_v_ currents of larger amplitude. We tested the possibility of removing the confounding effects contributed by other VGKC with fast-deactivating currents by analyzing the slow tail currents. Both mock- and *CLN1*-transfected cells generate slow tail currents at−50 mV with broadly similar kinetics and amplitudes, as shown for each cell by the cyan and blue sweeps nearly superimposing, a feature consistent with the expression of KCNH and KCNQ channels. To address this point, we measured the slowly decaying tail currents at−50 mV. Open and filled circles in panel 5D plot the fractional activation of tail currents at−50 mV in 15 mock and 13 *CLN1*-transfected cells, respectively. Average values of half-activation voltages generated by best-fits of the Boltzmann equation to conductance data of each cell indicate a significant difference between mock (-14.6 ± 3.8 mV, *N* = 15) and *CLN1* (-25.8 ± 3.2 mV, *N* = 13) (*t* = 2.1874 with 26 df, *P* = 0.0379 by two-tailed independent *t*-test). Normalized tail current amplitudes in panel E do not differ between mock (1.30 ± 0.26 pA/pF, *N* = 15) and *CLN1*-transfected cells (1.71 ± 0.26 pA/pF, *N* = 13) (*t* = 1.104 with 26 df, *P* = 0.2799 by independent *t*-test). One-way ANCOVA indicates a non-significant effect of either time after completing the differentiation [*F*_(1,24)_ = 0.02, *P* = 0.8887] or genotype [*F*_(1,25)_ = 1.09, *P* = 0.3065]. A similar analysis on half-activation voltages plotted in Figure 5F indicated that time in culture did not affect tail-currents half-activation voltages [*F*_(1,24)_ = 0.01, *P* = 0.9212], but confirmed the effect of genotype [*F*_(1,25)_ = 6.39, *P* = 0.0182 for genotype]. These results imply that, at any given time, tail currents of similar amplitude in mock- and *CLN1*-transfected cells flow through different combinations of Kv channels.

To address the contribution of different K_v_ channels to outward currents, we took advantage of the differential effects of 4-aminopyridine (4-AP), which blocks K_v_11 while upregulating K_v_12 channels (Elmedyb et al., 2007; Dierich et al., 2018) and does not affect K_v_7 channels (Khammy et al., 2018).

**Figure 6:**
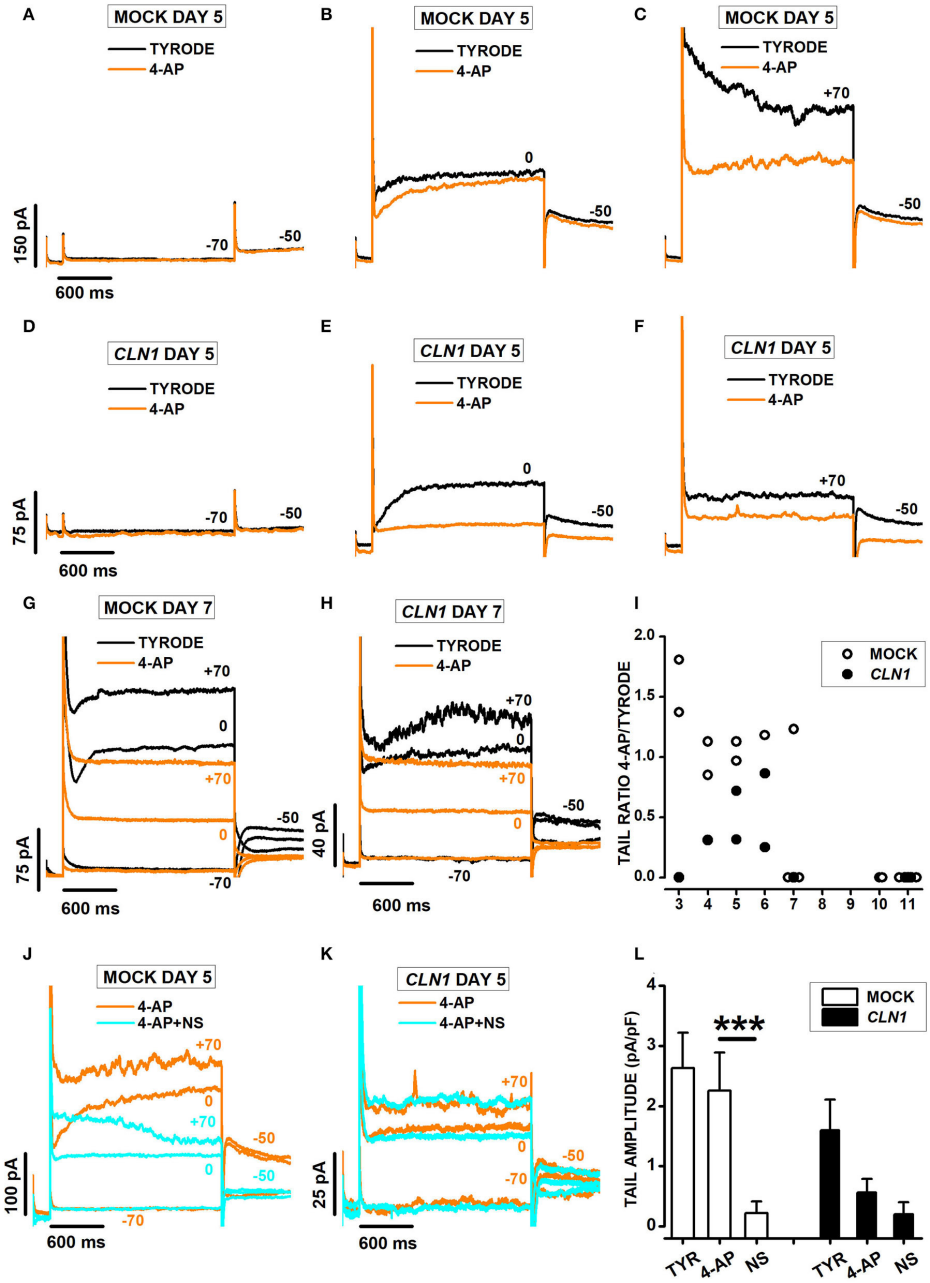
**(A–C)** Sweeps plot currents recorded 5 days after differentiation from a mock cell in response to a 2s-long voltage step−70 **(A)**, 0 **(B)**, and +70 **(C)** mV from a holding of −80 mV, in Tyrode (black) and Tyrode with 3 mM 4-AP (orange). **(D**–**F)** As for **(A–C)** above, but a *CLN1*-transfected cell. **(G,H)** Sweeps plot currents recorded 7 days after differentiation from a mock **(G)** and a *CLN1*-transfected **(H)** cell in response to 2s-long voltage steps to−70, 0, and +70 mV from a holding of −80 mV, in Tyrode (black) and Tyrode with 3 mM 4-AP (orange). **(I)** Data points plot ratios between tail currents recorded at −50 mV in the presence and in the absence of 4-AP as a function of time after differentiation for 14 mock (empty circles) and 10 *CLN1*-transfected (filled circles) cells. A two-way ANCOVA indicated significant effects of both genotype [*F*_(1,21)_ = 5.78, *P* = 0.0255] and time after differentiation [*F*_(1,20)_ = 9.06, *P* = 0.0069]. **(J,K)** Each panel plots currents recorded 5 days after differentiation in 3 mM 4-AP (orange) and 4-AP+30 μM NS-1643 (cyan) from the mock **(J)** and the *CLN1*-transfected **(K)** cells in **(A–C)** and **(E–G)**, respectively. **(L)** Columns plot average normalized tail current amplitudes, with their SEM, from 6 mock- (open bar) and 4 *CLN1*-transfected (filled bar) cells sequentially recorded in Tyrode (TYR), 4-AP, and 4-AP in combination with NS-1643 (NS). Two-way ANOVA for repeated measurements in time indicate significant effects for drugs [*F*_(2,16)_ = 24.95, *P* < 0.0001], but not genotype [*F*_(1,8)_ = 2.34, *P* = 0.1646]. The interaction between genotype and drug appears significant [*F*_(2,16)_ = 4.1, *P* = 0.0365]. ***P < 0.0001 by *post-hoc* analysis using a two-tailed paired *t*-test with Bonferroni correction for NS 1643 effect on tail amplitude in mock cells.

Currents recorded from mock and *CLN1* overexpressing cells showed opposing responses to the application of 3 mM 4-AP. Indeed, the drug did not reduce the amplitude of currents activated by voltage steps to−70 (Figure 6A) and 0 mV (Figure 6B) in the mock cell recorded 5 days after differentiation, with reduced amplitude at +70 (compare orange and black sweeps in Figure 6C). On the other hand, 4-AP (orange sweeps) strongly suppresses outward currents at both 0 and +70 mV in the *CLN1*-transfected cells (Figures 6E,F). Inspection of the representative records in panels B, C, E, and F revealed a striking difference between mock and SH-*CLN1* cells in the sensitivity of tail currents to 4-AP. The drug barely affected tail current amplitude in the mock cell, while almost entirely blocked them in the *CLN1* overexpressing cell. However, by day 7 after differentiation, tail currents of mock cells became sensitive to 4-AP, similar to *CLN1* overexpressing cells, as shown for two representative cells in panels 6G and 6H, respectively. Data in panel 6I plot the ratio between tail current amplitude in 4-AP and Tyrode for 14 mock (open symbols) and 10 *CLN1*-trasfected (filled symbols) cells. A two-way ANCOVA indicated significant effects of both genotype [*F*_(1,21)_ = 5.78, *P* = 0.0255] and time after differentiation [*F*_(1,20)_ = 9.06, *P* = 0.0069]. These data may indicate that the up-regulation of K_v_12 channels by 4-AP may mostly compensate the block of K_v_11 channels in mock cells up to 6 days after differentiation. In contrast, the reduced numbers of K_v_12 channels may prevent the compensation of blocked K_v_11 channels in *CLN1* overexpressing cells, and in mock cells since day seven after differentiation. To test this hypothesis, we used NS-1643 that, applied after the simultaneous block of K_v_11- and potentiation of K_v_12-mediated currents by 4-AP, effectively blocks currents flowing through K_v_12-channels. One-way ANCOVA did not find significant effects of both genotype [*F*_(1,21)_ = 1.69, *P* = 0.2077] and Cm [*F*_(1,20)_ = 0.09, *P* = 0.7673] on tail ratios (not shown).

As shown in Figures 6J–L, plotting sweeps recorded in the presence of either 3 mM 4-AP (orange) or the combination of 4-AP + NS-1643 (cyan). Note that NS-1643 nearly abolishes the tail currents at−50 mV in the mock-transfected cell (Figure 6J), but has marginal effects in the *CLN1* overexpressing cell (Figure 6K) whose tail currents resisted block by 4-AP. Columns in panel 6L plot average normalized tail current amplitudes, with their SEM, computed from 6 mock- and 4 *CLN1*-transfected cells sequentially recorded in Tyrode (TYR), 4-AP, and 4-AP in combination with NS-1643 (NS). Two-way ANOVA for repeated measurements in time indicates a non-significant difference between genotype [*F*_(1,8)_ = 2.34, *P* = 0.1646], consistent with the selection of *CLN1* cells whose tail currents resist block by 4-AP. However, the analysis indicates significant effects for both treatment [*F*_(2, 16)_ = 24.95, *P* < 0.0001] and the interaction between treatment and genotype [*F*_(2, 16)_ = 4.1, *P* = 0.0365]. *post-hoc* analysis by two-tailed paired *t*-test with Bonferroni correction for the number of contrasts indicates NS-1643 significantly reduces tail currents of mock cells recorded in 4-AP (*t* = 5.446 with 8 df, *P* < 0.001).

#### Expression of the VGKC Subunit KCNH4 (K_v_12.3)

**Figure 7:**
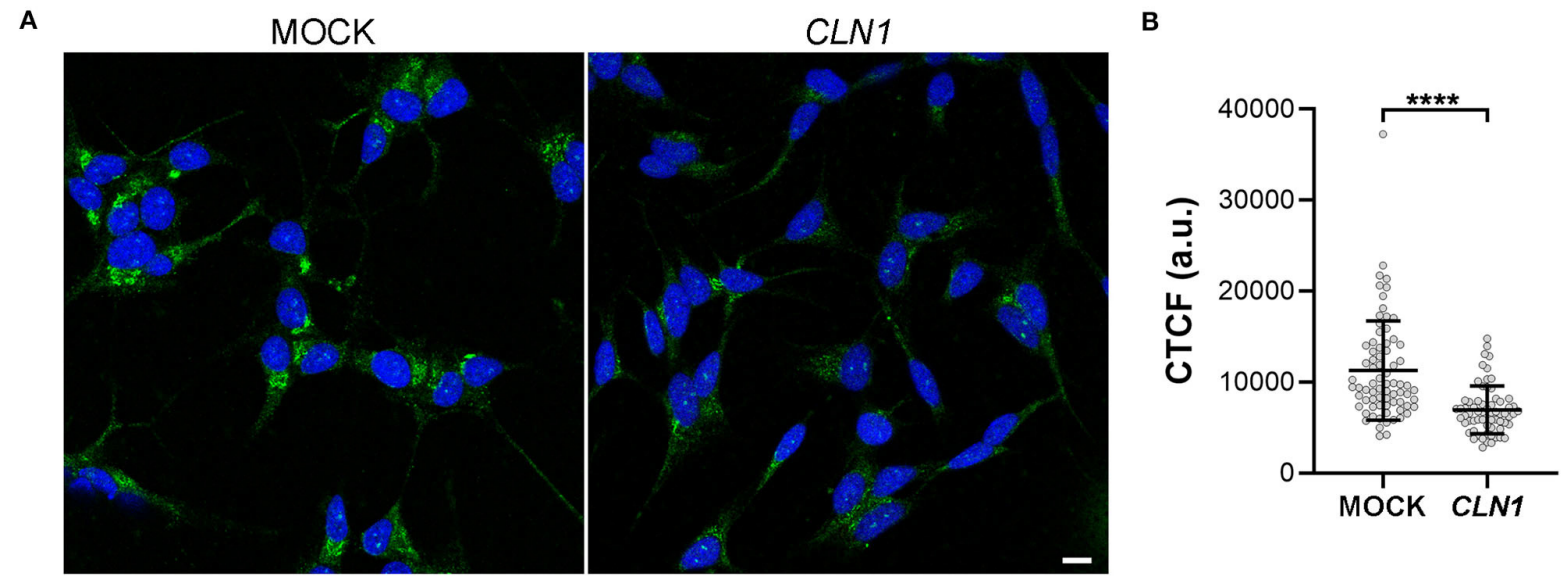
Immunofluorescence analysis of KCNH4 (Kv12.3) in differentiated mock- and *CLN1*-transfected cells. **(A)** Immunostaining by KCNH4 antibody revealed cytoplasmatic structures resembling ER cisternae in both differentiated mock- and *CLN1*-transfected cells. Bar equals to 10 μm. **(B)** Quantitative analysis of corrected cell fluorescence intensity (CTCF) by Fiji indicated a meaningful reduction of fluorescence signal in *CLN1*-transfected cells as compare to mock cells. ****P < 0.0001, Mann-Whitney *U*-test.

We then assessed the expression of a VGKC subunit coded by *KCNH4* (K_v_12.3), a DEGs of SH-*CLN1* transcriptomic profile. By using KCNH4 antibody, immunofluorescent assay revealed an ER pattern of staining shared between both differentiated mock and *CLN1*-trasfected cells (Figure 7A). Signal intensity was much lower in *CLN1*-trasfected cells as confirmed by CTCF quantitative analysis (Figure 7B).

### Palmitoylation Inquiry

Bioinformatic investigation through Swiss-Palm database (which collects proteomic studies and related palmitoylated identified proteins); (Blanc et al., 2019) was carried out to assess whether channel proteins encoded by DEGs of SH-*CLN1* may be putative targets of PPT1 *in vitro*. Considering DEGs enclosed in the three previously analyzed GO terms (namely *intrinsic component of synaptic membrane, voltage-gated ion channel activity*, and *modulation of chemical synaptic transmission*), only KCNK1 was found to be palmitoylated (see network in Supplementary Figure 2). However, the number of putative palmitoylated ion channels increased if one considers either palmitoylated ortholog proteins or the presence of cysteine residues, predicted *in silico* to be suitable for palmitoylation. In this view, CACNA2D3 and CACNG2 have orthologs reported to be palmitoylated. Likewise, CACNA2D2 and KCNH6 contain cysteine predicted to be modified by palmitoylation. Nevertheless, there is poor evidence supporting a modification through palmitoylation for most of the ion channels coded by DEGs identified in *CLN1* overexpressing cells.

## Discussion

Bioinformatic surveys of the transcriptomic profile of SH-SY5Y cells, overexpressing *CLN1* and differentiated into a neuronal-like phenotype, disclosed networks of genes involved in two main neuronal functional modules, namely the acquisition of morphological neuronal-like features and synaptic compartment and functioning (Pezzini et al., 2017b). These puzzling findings were not detected in the same cellular system over-expressing the most frequent Italian variant of *CLN1* (i.e., c.665T>C), and may indicate that the described events are obtained when the fully-functional protein is over-expressed, whereas a mutated protein (no matter which kind of mutations) is “silenced” if overexpressed (Pezzini et al., 2017b). In this study, we focused our attention on the dysregulation of genes coding for subunits of voltage-gated ion channels (either VGCC or K_v_, see Table 1). Results show that *CLN1* overexpression induces the remodeling of the electrophysiological profile of the human neuroblastoma-derived cell line SH-SY5Y by the selective suppression of a subset of voltage-gated calcium and potassium currents. The notion that an increase in PPT1 affects channel proteins outside the human palmitoylome represents a significant conceptual advancement toward the identification of PPT1 mechanisms of actions in nerve cells. We are aware of the limitations of this experimental setting as far CLN1 disease is concerned; therefore studies on a KO cellular model (using the SH-SY5Y cells) following a similar methodological approach are in progress in our laboratories: transcriptomic analysis will lead us to perform selected functional, biochemical (proteomic) and cell physiology studies matching the putative results with those obtained in our previous studies (Scifo et al., 2015; Pezzini et al., 2017b) and in the present one.

### Electrophysiological Properties of Differentiated SH-SY5Y Cells

Chemical cues, provided by RA-NBM, promote SH-SY5Y differentiation toward a neuronal phenotype, characterized by shield-like morphology of their cell body and a long axon that express synaptic markers (Encinas et al., 2000; Pezzini et al., 2017a). Previous electrophysiological analysis indicated a considerable variability between the electrophysiological profiles of undifferentiated SH-SY5Y (Santillo et al., 2014). Patch-clamp and calcium imaging data from this study have shown that most mock SH-SY5Y cells generate voltage-gated calcium and potassium currents upon culturing in neuronal differentiation medium, as expected from adult neurons, and therefore indicate they are suitable for electrophysiological studies. The slow kinetics of currents and the sensitivity to drugs such as 4-AP and NS-1643, all agree with the expression of K_v_12 channels by differentiated SH-SY5Y cells. The notion of membrane currents through K_v_12 channels is intriguing, considering their expression pattern, somewhat restricted to telencephalic/diencephalic neurons (Zou et al., 2003). Likewise, the expression of additional members of the K_v_11 subfamily (i.e., KCNH2 and KCNH6) also suggests that chemical cues promote, in a subset of SH-SY5Y cells at least, the development of functional properties consistent with a neuronal-like phenotype. Altogether these findings sustain the successful neuronal differentiation of SH-SY5Y cells in such experimental setting. Intriguingly, we found that mock cells progressively change some of their functional features within a few days after switching from the RA containing medium to enriched NBM lacking RA. Specifically, data on K_v_ currents suggest a progressive loss of K_v_12 channels, with mock cells lacking functional K_v_12 channels after days 7 from RA-containing medium removal. However, the loss of K_v_ channels, does occur in a selective way, with cells keeping the expression of functional calcium channels, according to both patch-clamp and calcium imaging data, up to 10 days after switching in enriched NBM medium. The mechanisms underlying the differential regulation of calcium and K_v_12 channels by RA-containing medium components are presently unknown. Beside the roles of specific components, it is possible that differentiation does not occur in a stepwise mode, and different ion channels may not start their expression in a synchronous way. Differentiation may rather occur as a sequence of steps spaced in time by tightly-controlled delays. In this case, earliest differentiation events may last longer than those occurring at later times. In the limited time span of our study, we may well find the loss of late-occurring differentiation steps (i.e. the expression of K_v_12 channels), but fail to recognize the loss of early-occurring steps (i.e. the expression of calcium channels). Future experiments monitoring the functional expression of ion channels at earlier differentiation times in RA-containing medium may address this point, along with data collection at longer times after completing the differentiation protocol. It is also relevant to note that the remodeling of KCNH subunits with time after differentiation in mock cells does not occur at the expense of tail currents amplitudes, which remain of similar amplitudes in both mock and *CLN1*-overexpressing cells. This observation may indicate that KCNH targeting to the membrane reflect a role for RA-medium specific factors in promoting isoform-independent trafficking, similar to the non-significant decrease of membrane conductance in the range−90/-70 mV with time after differentiation.

### Electrophysiological Profiles of Differentiated SH-*CLN1* Cells

Results on normalized membrane conductance provide independent support to the notion that *CLN1* overexpression does not affect plasma membrane characteristics in a range of membrane potentials close to resting values. Membrane potentials close to the Nernst potential for potassium ions, in both mock and *CLN1*-transfected cells, are in accordance with the same notion (Figure 1). Nevertheless, the number of functional calcium channels reaching the plasma membrane is substantially reduced in *CLN1*-transfected cells, as revealed by both patch-clamp and calcium imaging data following appropriate stimuli (Figures 2, 3). Neither time after differentiation, nor membrane capacitance affects the impact of charge carrier on inward current amplitudes. However, the ANCOVA, using as a covariate the membrane capacitance (an index of plasma membrane surface) indicates Cm reduces the effect of genotype. Considering that membrane currents were expressed as current densities by normalization to the cell capacitance, this may indicate that cell surface independently affects the number of channels reaching the plasma membrane. Indeed, *CLN1*-transfected cells appear to have less elaborated axonal branching, which may reflect a shortage of sites for channels insertion. However, immunostaining in SH-SY5Y cells does not have enough sensitivity to reveal axonal hot spots in either mock or *CLN1* overexpressing cells to support this hypothesis.

*CLN1* overexpression also significantly reduces the number of a subset of functional VGKC in the plasma membrane of differentiated SH-SY5Y cells. The functional properties and the sensitivity to drugs of these channels are consistent with reduced K_v_12. Altogether, these observations indicate that an increased PPT1 activity triggers the remodeling of the electrophysiological profile of SH-SY5Y cells by selectively affecting two distinct families of VGIC. Data from tail current analysis reveal some intriguing features of *CLN1* overexpression on KCNH channels regulation. The finding that *CLN1* overexpression does not change tail current amplitude may indicate that the number of KCNH channels reaching the plasma membrane is tightly controlled. Expression data indicate KCNH2 and KCNH6 (K_v_11.1 and K_v_11.2) as the KCNH isoforms with the highest expression in both mock and *CLN1*-transfected cells. Assuming higher gene expression translates into higher protein levels, which would in turn increase the chance of accessing the trafficking machinery, we would expect K_v_11 channels as the prevailing currents in both mock- and *CLN1*-transfected cells. In contrast, data in Figure 6 indicate that the K_v_11 channels block by 4-AP does not affect tail current amplitudes in mock cells up to 7 days after completing the differentiation protocol, while strongly suppressing tail currents in *CLN1*-transfected cells at all times after differentiation. These findings may indicate that in mock cells K_v_12 channels may have a preferential access to the trafficking machinery, thus overcoming K_v_11 channels higher expression. This possibility may require further investigation to assess the correlation between K_v_11 protein and gene expression levels in both mock and *CLN1*-overexpressing cells. A different possibility would be that *CLN1* overexpression reduces K_v_11 stability in the plasma membrane, ending up with K_v_12 accumulation. However, IF data showing reduced KCNH4 (K_v_12.3) staining in *CLN1* overexpressing cells does not support a mechanism primarily based on reduced K_v_11 stability.

### Correlations Between Bioinformatic Data and Molecular Findings

The evidence of the expression of selected ion channels (*CACNA2D2, KCNH4*, and *KCNH6*) by SH-SY5Y cells lays the ground for the electrophysiological study. A relationship between transcriptomic results and protein expression data was detected for *CACNA2D2* only. Downregulation of the transcript (log_2_FC = −1.11) in differentiated *CLN1* overexpressing cells was associated with lower expression of two isoforms of CACNA2D2 (and especially of the 170/140 kDa isoform) in homogenates of the same cell line, as compared to differentiated mock cells (Figure 4A and Supplementary Figure 4). Interestingly, the 170 kDa isoform likely corresponds to the mature, glycosylated protein exposed to cell surface, which provides the structural elements required for channel stimulation [Figure 4C and data in Gurnett et al. (1996), Hoppa et al. (2012)]. The 85 kDa immunoreactive band may represent an intermediate glycosylated isoform of CACNA2D2 expressed by SH-SY5Y or a degradation product generated during the harvest of cells. In our view, the biochemical findings suggest that intracellular processing of CACNA2D2 and its translocation to the outer membrane surface of the cell occurred properly even in differentiated *CLN1* overexpressing cells, although less amount of mature protein may reach the plasma membrane. Altogether these data are in agreement with the biochemical properties of CACNA2D2/α_2_δ-2 previously reported in different cellular settings (Davies et al., 2010; Neely and Hidalgo, 2014; Dolphin, 2016, 2018; Tedeschi et al., 2016) and consistent with its expression in SH-SY5Y cells.

As for VGKC, our cell physiology data demonstrated the decrease in the number of functional channels, whose properties are consistent with K_v_12 channels, reaching the plasma membrane upon *CLN1* overexpression. The investigation of the *KCNH4* was partially achieved by the IF assay, which pinpointed a decreased fluorescence intensity of KCNH4 immunostaining *CLN1*-transfected cells. These findings are consistent with the marked expression of the protein in the ER, whereas an evidence of translocation to the cell membrane has not been demonstrated. However, the differences in IF intensity are consistent with the results of RNAseq analysis.

### Putatively Impaired Cellular Mechanisms

The observed correlations between bioinformatic data and cell physiology study raise, however, several questions as for the pathomechanisms leading to ionic channel malfunctioning in this experimental setting. It is tempting to hypothesize that increased PPT1 levels may hamper the functioning of several subunits of ion channels by changing their palmitoylation state (Shipston, 2014).

Recent findings pinpointed a molecular link between PPT1, CSPα/DNAJC5 and some potassium channels. CSPα is a palmitoylated co-chaperone protein of synaptic vesicles: mutations in its cysteine string domain lead to an adult-onset form of Neuronal Ceroid Lipofuscinosis, CLN4 (Nosková et al., 2011; Zhang and Chandra, 2014; Henderson et al., 2016). Alterations in CSPα affects the expression of a subset of palmitoylated K^+^ channels, namely large-conductance calcium and voltage-gated potassium (BK) channels, and their related currents (Jeffries et al., 2010; Kyle et al., 2013; Donnelier et al., 2015). As discussed in point 4.2 above, the notion that the altered palmitoylation state of a chaperone protein (along with mis-localization and accumulation of PPT1) impairs the trafficking to the plasma membrane of voltage-gated potassium channels bears a clear relevance to our results.

*In silico* prediction suggested that several of the channel proteins identified in our cell model may be palmitoylated. For instance, CACNA2D2/α_2_δ-2 and CACNA2D3/α_2_δ-3 contain cysteines or have an orthologous protein which has been identified as palmitoylated (Blanc et al., 2019). Interestingly, these two α_2_δ proteins have been affinity purified from a cortical mouse lysate by using a recombinant PPT1 enzyme, and an altered amount of CACNA2D2 was also detected by a proteomic approach (Sapir et al., 2019). We cannot rule out that the observed malfunctioning of calcium channels may be derived from an incorrect processing of α_2_δ-2, necessary to drive mature channels toward plasma membrane domain (Gao et al., 2000; Dolphin, 2016; Kadurin et al., 2016; Ferron et al., 2018).

Lastly, it remains unsolved the full chain of events in CLN1 disease. One might argue whether the reduced gene expression represents the consequence rather than the cause of the defective trafficking of channels. *CLN1* overexpression may lead to accumulation of stacked immature channel proteins, triggering a feedback mechanism that would switch-off the transcription of VGIC genes.

However, these issues deserve targeted investigations, which were beyond the purpose of this study.

### Concluding Remarks

Differentiated SH-SY5Y cells, which present a neuronal-like phenotype, represent a suitable model for electrophysiological studies *in vitro*. Overall, data from this study have shown that *CLN1* overexpression in these cells is associated with profoundly reduced expression of transcripts coding for voltage-gated calcium and potassium channel subunits. That may provide some clues as for a potential role of these genes in the pathogenetic mechanisms related to CLN1 disease.

**Figure 8:**
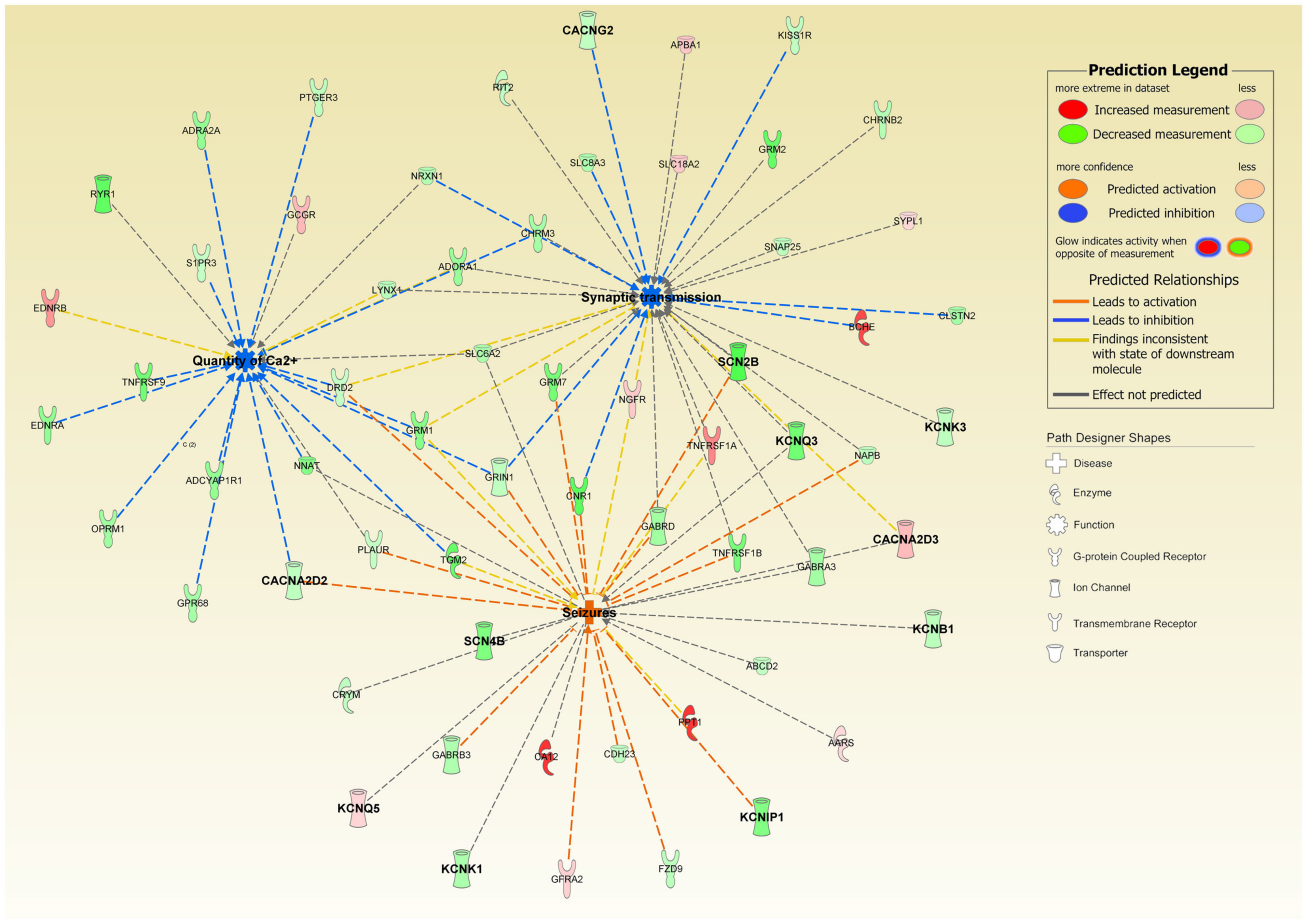
Ingenuity Pathway Analysis of DEGs associated with the overexpression of *CLN1*-transfected cells revealed a significant involvement of functional annotations related to “*Quantity of Calcium*” (predicted inhibited, z-score = −3.108) and “*Synaptic Transmission*” (predicted inhibited, z-score = −1.279); interestingly, “*Seizures*” annotation was also predicted to be activated in our experimental settings (z-score = 1.951). Genes coding for Calcium and Potassium ion channels are in bold. Notably, CACNA2D2 bridges “*Quantity of Calcium*” and “*Seizures*” and supports their predicted activation state.

Patch-clamp profiles of differentiated SH-SY5Y cells have shown that *CLN1* overexpression is associated with selective remodeling of the electrophysiological properties of these cells; likewise, calcium imaging studies revealed reduced Ca2+ influx in accordance with the bioinformatic prediction of CACNA2D2 involvement in this experimental setting. These mechanisms may be related to and facilitate an onset and maintenance of the untreatable seizures observed in CLN1 disease as highlighted by the putative activating role of CACNA2D2 on seizures (Figure 8). These findings require more targeted experimental work using other cellular systems. Not much is known of the electrophysiological properties and seizure activity in *CLN1* KO mouse models. However, a protective role of PPT1 from excitotoxicity was suggested in induced status epilepticus in rat (Suopanki et al., 2002). Focusing on abnormal currents and related VGICs investigated in this *CLN1* overexpression study may also contribute to better understanding the pathomechanisms underlying the untreatable epilepsy present in CLN1 disease (Vanhanen et al., 1997).

The biochemical characterization of α_2_δ-2 did not reveal post-translational changes of the protein, but decreased amounts of the expressed protein in the homogenates of differentiated cells overexpressing *CLN1* in line with its reduced mRNA expression. Likewise, the negative effect on axonal growth of *CLN1* overexpression in SH-SY5Y cells leading to reduced neuronal plasma membrane surface, may result in less protein translocated to the membrane, and it does not seem to be directly related to changes in *CACNA2D2* expression. *CACNA2D2* is involved in axonal growth and branching in an age regulated manner in murine dorsal ganglia neurons. Namely, *CACNA2D2* overexpression is associated with inhibition of axonal growth and elongation, whereas a 2 bp deletion of the gene in the ducky-2j mutant mouse promotes axonal growth and affects collateral branching (Tedeschi et al., 2016). Another major function ascribed to α_2_δ subunits (including α_2_δ-2) is to modulate the differentiation of presynaptic endings (Dickman et al., 2008; Kurshan et al., 2009), and to induce the formation of synapses both *in vitro* and *in vivo* (Eroglu et al., 2009). Altogether, data from the literature indicate a relevant role for α_2_δ-2 in axonal compartments (such as the synaptic junctions at the nerve endings and the plasma membrane), which are primarily involved in the electrical activities of neurons. It is unclear if the impaired electrical activity associated with reduced α_2_δ-2 expression in SH-SY5Y overexpressing *CLN1* relates to the abnormal firing of neuronal cells in other experimental settings. It is worth remarking, however, that biallelic variants in *CACNA2D2* are detected in an early onset form of epileptic encephalopathy, which shares some clinical features with CLN1 disease (Brill et al., 2004; Ivanov et al., 2004; Punetha et al., 2019). Recent evidence suggests that the effects of *CACNA2D2* on axonal growth and elongation as well as on synaptic formation are inhibited by pregabalin, a drug which targets the α_2_δ-2 subunit and which is used in several neurological conditions, including epilepsy (Tedeschi et al., 2016). Testing the efficacy of this drug in cellular and animal models of PPT1 deficiency might become a step forward to cope with severe neuronal dysfunction observed in CLN1 disease. Among open questions to be answered remain the mechanisms leading to the remodeling of functional channels on the plasma membrane associated with overexpressed *CLN1* and the putative role *CACNA2D2* on neuronal dysfunction and pathology in CLN1 disease.

## Data Availability Statement

The datasets presented in this study can be found in online repositories. The names of the repository/repositories and accession number(s) can be found at: NCBI [accession number: GSE98834].

## Author Contributions

GD, FP, FS, and AS conceived and designed the study and wrote and drafted the manuscript. GD and EM performed patch clamp and electrophysiological investigations. GD, EM, and BL performed the Ca^2+^ flux imaging. FP and MB performed transfection experiments. FP performed biochemical and morphological investigations. FP and SD performed bioinformatic analysis. GD, ML, FS, and AS checked the accuracy of data, the appropriateness of investigations, and gave the final approval of the manuscript for publication. All authors contributed to the article and approved the submitted version.

## Conflict of Interest

The authors declare that the research was conducted in the absence of any commercial or financial relationships that could be construed as a potential conflict of interest.

## Abbreviations

4-AP: 4-aminopyridine
ΔF/F0: fluorescence normalized to basal fluorescence
Cm: membrane capacitances
DEG: Differentially Expressed Gene
FDR: False Discovery Rate
FPKM: Fragments Per Kilobase per Million mapped reads
log_2_FC: Log_2_ fold change
PPT1: Palmitoyl-Protein Thioesterase 1
PK: Proteinase K
RA-NBM: differentiation medium (all*-trans-*Retinoic Acid and enriched Neurobasal medium)
SH-mock: pcDNA3 (empty vector) transfected cell line
SH-*CLN1*: *CLN1*-transfected cell line
VGCC: Voltage-gated Calcium Channels
VGKC (or K_v_): Voltage-gated Potassium Channels
VGIC: Voltage-gated Ion Channels.
