# Supplementary material for: Electrophysiological Profile Remodeling via Selective Suppression of Voltage-Gated Currents by CLN1/PPT1 Overexpression in Human Neuronal-Like Cells
Source: Front Cell Neurosci. 2020 Dec 16;14:569598. doi: 10.3389/fncel.2020.569598 (PMC7772423; doi:10.3389/fncel.2020.569598)
Supplement: Supplementary file 3 [file Data_Sheet_1.PDF]

frontiers

Supplementary Material

1 Supplementary Data

1.1 Bioinformatic Data

Further data of transcriptomic analysis of SH-*CLN1* cells are reported in Supplementary Table S1 and S2 and described here below.

1.2 VGCC coding genes

Voltage-gated calcium channel (VGCC) gene family is composed by 24 genes, encompassing four sub-groups, namely α1 (n=10), β (n=4), $\alpha_2\delta$ (n=4) and γ (n=8) subunits. By applying a threshold for FPKM at least > 0.5, we found that differentiated (mock and SH-*CLN1*) cell lines expressed most of these genes; however, DEGs identified in SH-*CLN1* belonged only to $\alpha_2\delta$ and γ subfamilies. Of particular interest were the two DEGs coding for $\alpha_2\delta$ subunits (*CACNA2D2* and *CACN2D3*), because they are two dysregulated genes out of three genes expressed in our cell system. The other DEG of VGCC family was *CACNG2*, coding for the auxiliary subunit gamma 2 (also known as TARP-γ) which was found significantly down-regulated. No significant changes in the expression of α1 subunits or auxiliary β subunits encoding genes were observed, even though several genes were very close to the thresholds utilized for the assignment of DEGs. For instance, *CACNA1B*, the N-type isoform $Ca_v2.2$ of VGCC involved in neurotransmitter release, as well as *CACNA1C* was found to be down-regulated (log_2FC=-1.33 and -1.53, respectively), but their q-values were not significant. Conversely *CACNA1D* and *CACNA1G* showed a significant q-values, but the associated fold changes were slightly up-regulated (log_2FC=0.87 and 0.97, respectively). In addition, an up-regulation of *CACNB2* (log_2FC=1.01), encoding for the cytoplasmic β2 accessory subunit, was seen, but without a significant q-value (>0.05).

1.3 Potassium Channels coding genes

Potassium Channel gene family encompasses 79 members of which 41 were found in the transcriptomes of differentiated SH-mock and SH-*CLN1* cell lines (considering a FPKM at least > 0.5; Supplementary Table S2). In this view, the subset of 10 DEGs of SH-*CLN1* transcriptomic profile corresponds approximately to the 25% of total number of genes encoding potassium channels expressed in this cell system. Moreover, 6 DEGs belong to Voltage-gated Potassium Channel (VGPC or Kv) subfamily, which was also the most represented group of genes expressed in our cell system.

In general, most potassium channels-coding genes are downregulated in *CLN1*-overexpressing cells, except for *KCNQ5*, which shows a reverse pattern (i.e. an increased expression as compared to mock cells). Among DEGs encoding for K_v, of particular significance was the downregulation of *KCNH4* and *KCNH6*, which code for $K_v12.3$ and $K_v11.2$ respectively. Other relevant genes belonging to the same subfamilies were *KCNH2* and *KCNH8*, respectively. *KCNH2* (coding for $K_v11.1$, a member of

the KCNH [eag] family of potassium channels), is the gene with the highest expression in both differentiated cell lines, and it was similarly expressed in SH-*CLN1* and mock cells. In addition, one DEG of SH-*CLN1* profile (KCNIP1) encodes for a cytosolic K_v-interacting proteins (KCNIPs), regulating the activity of K_v4.3 and possibly the GABAergic transmission (Bourdeau et al, 2011; Del Pino et al, 2015).

1.4 Supplementary References

Bourdeau ML, Laplante I, Laurent CE, and Lacaille J-C (2011). KChIP1 modulation of Kv4.3-mediated A-type K+ currents and repetitive firing in hippocampal interneurons. *Neuroscience*. 176:173-87. doi: 10.1016/j.neuroscience.2010.11.051.

Del Pino J, Frejo MT, Baselga MJ, Capo MA, Moyano P, García JM, and Díaz MJ (2015). Neuroprotective or neurotoxic effects of 4-aminopyridine mediated by KChIP1 regulation through adjustment of Kv 4.3 potassium channels expression and GABA-mediated transmission in primary hippocampal cells. *Toxicology*. 333:107-117. doi: 10.1016/j.tox.2015.04.013.

2 Supplementary Figures

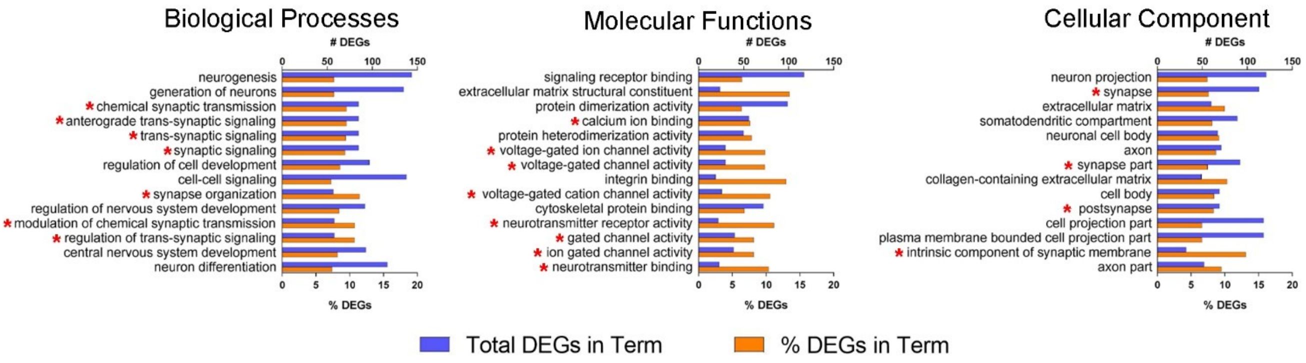

Supplementary Figure S1.

Enrichment analysis of DEGs identified in *CLN1*-overexpressing cells by ToppFun. Many GO terms are associated with synaptic compartment, neurotransmission and voltage gated channels (marked by asterisks). It is noteworthy that the percentage of DEGs on the total number of genes enclosed in the GO term is particularly higher in annotations related to voltage-gated channels (orange bar).

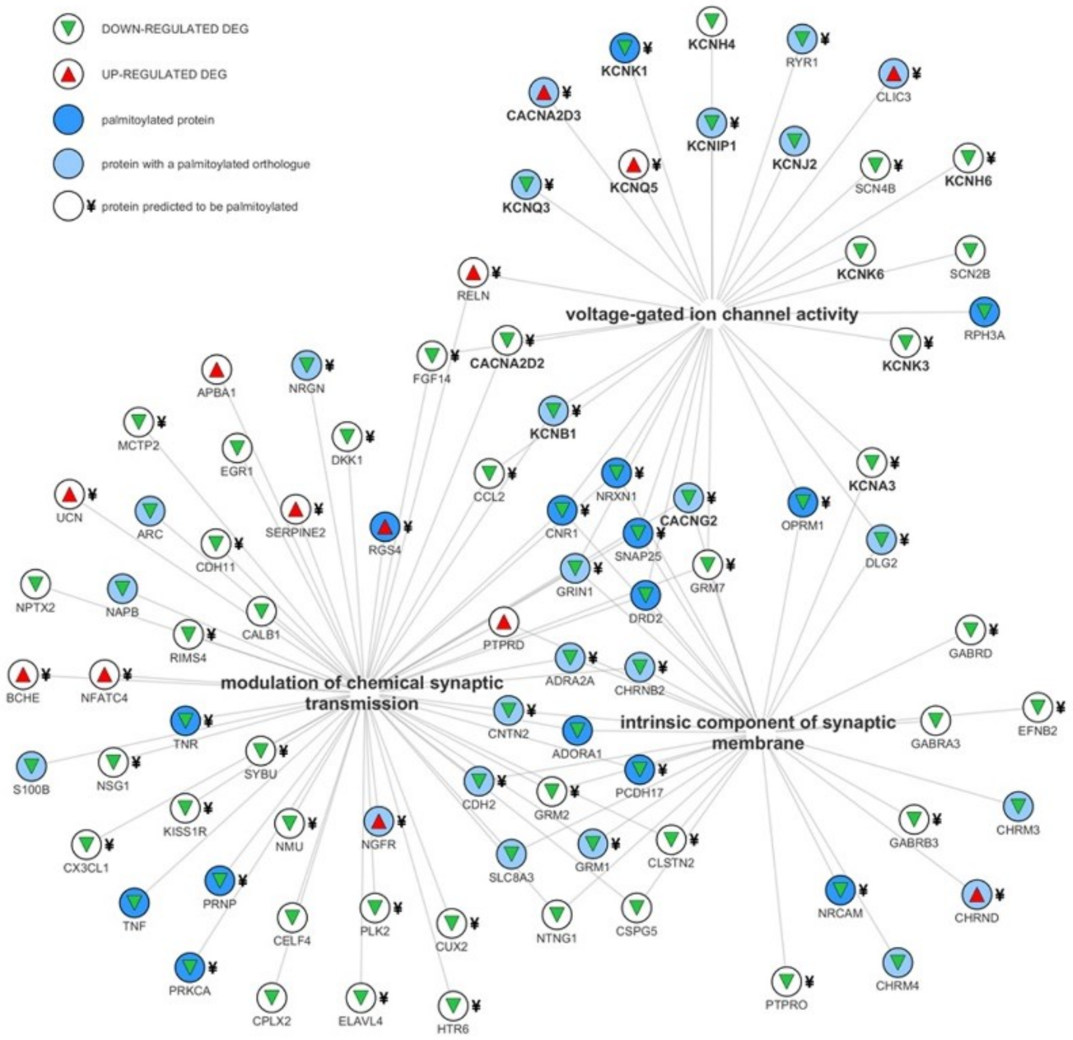

Supplementary Figure S2.

Network of DEGs enclosed in three selected GO terms (*intrinsic component of synaptic membrane* [GO:Cellular Component, 0099240]; *voltage-gated ion channel activity* [GO: Molecular Function, 0005244] and *modulation of chemical synaptic transmission* [GO: Biological Process, 0050804]). Genes coding for Calcium or Potassium channels are in bold. Genes which encode for palmitoylated proteins or which have a palmitoylated orthologue are reported in blue and cyan respectively; genes coding for proteins predicted to be palmitoylated are marked by ¥.

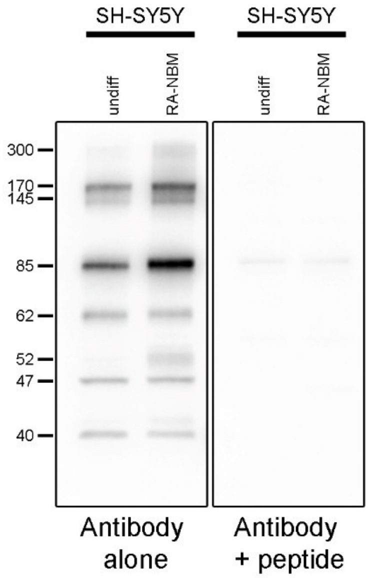

Supplementary Figure S3.

Pre-incubation assay to test the specificity of the immunolabelling using rabbit polyclonal antibody (Alomone, 1:2000). Immunoblotting of SH-SY5Y cells under basal conditions and following RA-NBM differentiation reveals two 170/140 and 85 kDa bands, corresponding to predicted isoforms of CACNA2D2. Faint bands of lower molecular weights are also detected (left lanes). No immunolabelling is observed following pre-absorption with the corresponding antigenic peptide (right lanes).

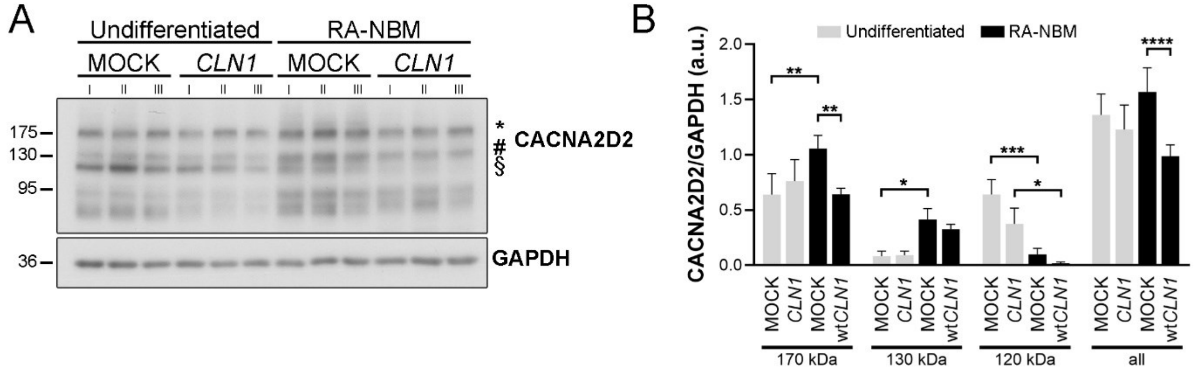

Supplementary Figure S4.

Immunoblotting investigation of the expression of CACNA2D2 by using the rabbit polyclonal antibody purchased from Biorbyt, which recognizes the ammino acids 19-30 at the N-terminus of the protein. **(A)** Three isoforms of CACNA2D2, running approximately at ~170 kDa (marked by *), ~130 kDa (#) and ~120 kDa (§) were detected by the antibody; these bands may represent the pro-protein (~130 kDa), the α_2 chain (~120 kDa) and the mature form $\alpha_2\delta 2$ (~170 kDa). Other bands running approximately at 95-80 kDa were also detected, similarly as by using the Alomone polyclonal antibody. The expression of the three bands, especially of the 130 and 120 kDa bands, changed following neuronal differentiation in RA-NBM medium, suggesting a modulation of CACNA2D2 isoforms under differentiating conditions. **(B)** Densitometric semi-quantitative analysis indicated a significant decreased amount of 170 kDa isoform in *CLN1*-overexpressing cells, following neuronal differentiation; considering the expression of all three isoforms, the difference between mock and SH-*CLN1* cells was more evident (bars named "all"). Two-way ANOVA followed by Tukey's multiple comparisons test; * $P<0.05$, ** $P<0.01$, *** $P<0.001$, **** $P<0.0001$. I, II, III represent cellular homogenates from three independent differentiation experiments.

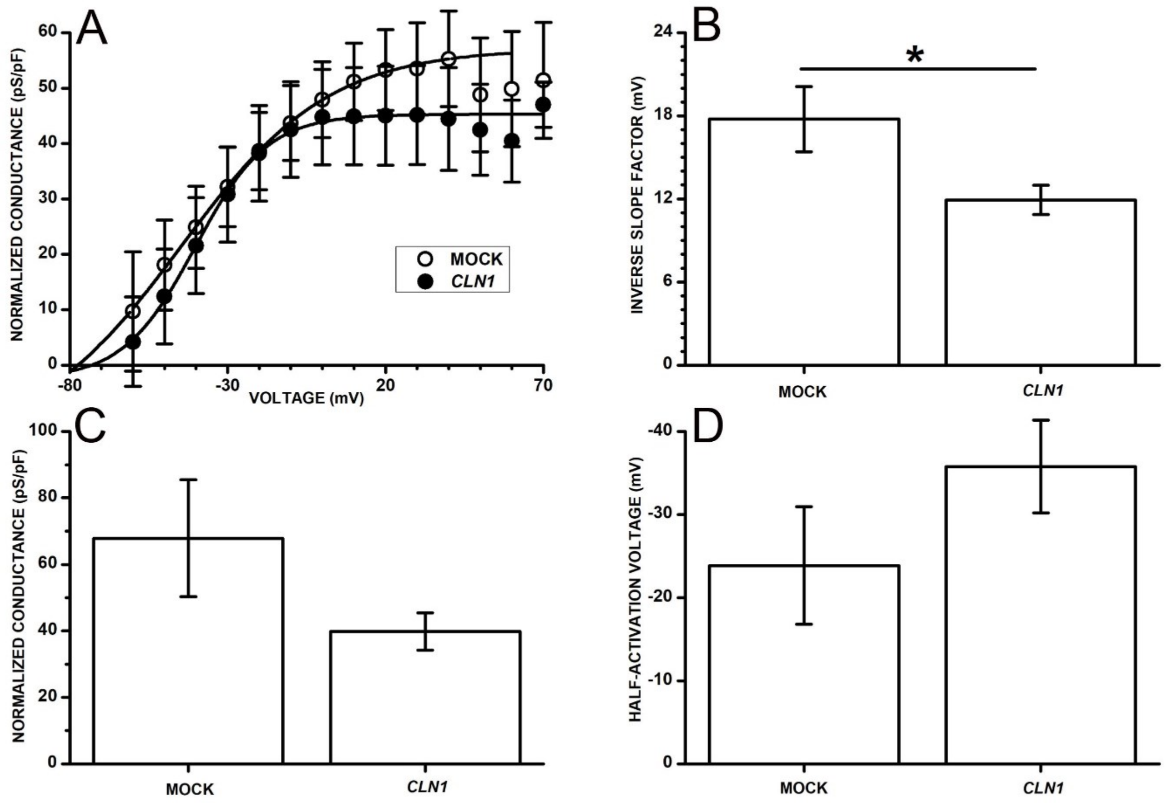

Supplementary Figure S5

(A) Data plot average normalized conductance with their SEM for mock (open circles, N=15) and *CLN1*-transfected (filled circles, N=14) cells, along with best-fitting Boltzmann equations. **(B)** Data plot average ±SEM inverse slope factors (S) for *CLN1*-transfected cells (filled circles) (11.92±1.06 mV) and mock cells (17.77±2.36 mV) (t=2,206 with 27 df, *P*=0.036 by independent t-test). **(C)** Average maximal normalized conductance data for mock (67.89±17.59 pS/pF) (open circles) and *CLN1*-transfected cells (39.81±5.58 pS/pF) filled circles do not differ significantly (t=1,478 with 27 df, *P*=0.151 by two-tailed independent t-test). **(D)** Average half-activation voltages of mock (-23.87±7.07 mV) (open circles) and *CLN1*-transfected cells (-35.79±3.96 mV) (filled circles) do not differ significantly (t=1.441 with 27 df, *P*=0.161). One-way ANCOVA indicates a lack of significant effect for both genotype and days after differentiation on maximal conductance (genotype $F_{1,26}$=2.26, *P*=0.1448; days after differentiation $F_{1,25}$=2.63, *P*=0.1174) and half-activation voltage (genotype $F_{1,26}$=1.95, *P*=0.1744; days after differentiation $F_{1,25}$=0.03, *P*=0.8639). Conductance was generated from total membrane current (I_{tot}) by assuming I_{tot} is the sum of a linear component (I_{Leak}) and a voltage-dependent component (I_V): $I_{tot} = I_{Leak} + I_V$

Subtracting I_{Leak}, extrapolated to every potential from a linear fit of membrane current at -90, -80, and -70 mV, from I_{tot} returns I_V, which is converted into conductance by dividing for the potassium driving force (V-EK): $\frac{I_V}{V-E_K} = g_V$

g_V is then fitted by Eq.2 in the methods section to generate G_{max}, V0.5, and S.
